# Characterization and Function of 3-Hydroxy-3-Methylglutaryl-CoA Reductase in *Populus trichocarpa*: Overexpression of *PtHMGR* Enhances Terpenoids in Transgenic Poplar

**DOI:** 10.3389/fpls.2019.01476

**Published:** 2019-11-15

**Authors:** Hui Wei, Chen Xu, Ali Movahedi, Weibo Sun, Dawei Li, Qiang Zhuge

**Affiliations:** ^1^Co-Innovation Center for Sustainable Forestry in Southern China, Key Laboratory of Forest Genetics & Biotechnology, Ministry of Education, College of Biology and the Environment, Nanjing Forestry University, Nanjing, China; ^2^Jiangsu Provincial Key Construction Laboratory of Special Biomass Resource Utilization, Nanjing Xiaozhuang University, Nanjing, China

**Keywords:** carotene, lycopene, Populus trichocarpa, *PtHMGR*, terpenoid

## Abstract

In the mevalonic acid (MVA) pathway, 3-hydroxy-3-methylglutaryl-CoA reductase (HMGR) is considered the first rate-limiting enzyme in isoprenoid biosynthesis. In this study, we cloned a full-length cDNA from *Populus trichocarpa* with an open reading frame of 1,734 bp. The deduced PtHMGR sequence contained two HMG-CoA motifs and two NADPH motifs, which exhibited homology with HMGR proteins from other species. Subsequently, truncated PtHMGR was expressed in *Escherichia coli* BL21 (DE3) cells, and enzyme activity analysis revealed that the truncated PtHMGR protein could catalyze the reaction of HMG-CoA and NADPH to form MVA. Relative expression analysis suggests that *PtHMGR* expression varies among tissues and that *PtHMGR* responds significantly to abscisic acid (ABA), NaCl, PEG_6000_, hydrogen peroxide (H_2_O_2_), and cold stresses. We used polymerase chain reaction (PCR) analysis to select transgenic Nanlin 895 poplars (*Populus× euramericana* cv.) and quantitative reverse-transcription PCR (qRT-PCR) to show that *PtHMGR* expression levels were 3- to 10-fold higher in transgenic lines than in wild-type (WT) poplars. qRT-PCR was also used to determine transcript levels of methylerythritol phosphate (MEP)-, MVA-, and downstream-related genes, indicating that overexpression of *PtHMGR* not only affects expression levels of MVA-related genes, but also those of MEP-related genes. We also measured the content of terpenoids including ABA, gibberellic acid (GA), carotenes, and lycopene. *PtHMGR* overexpression significantly increased ABA, GA, carotene, and lycopene content, indicating that PtHMGR participates in the regulation of terpenoid compound synthesis.

## Introduction

Isoprenoids (or terpenoids) represent a large and diverse group of primary and secondary metabolites present in all living organisms. For example, sterols are essential primary isoprenoids that are the main components of biomembranes. Chlorophylls and carotenoids are important photosynthetic pigments. Several isoprenoid-derived plant hormones regulate plant growth, development, and defense against biotic and abiotic stresses (Bouvier et al., 2005; Kirby and Keasling, 2009). As important biological compounds, isoprenoids participate in an array of specialized biological processes of fundamental adaptive or regulatory importance at the levels of individual cells, tissues, and whole organisms (Eisenreich et al., 2001; Grassmann et al., 2002). Isoprenoids originate from isopentenyl diphosphate (IPP) and dimethylallyl diphosphate (DMAPP), and include carotenoids, phytoalexins, phytosterols, specific terpenoids, growth hormones, and polyprenols such as dolichols (oxidized derivatives of polyprenols of a certain chain length), quinones, and isoprenoid conjugates of proteins (Hunter, 2007; Suzuki and Muranaka, 2007; Cao et al., 2010). Previous studies have identified two distinct isoprenoid biosynthesis pathways in plants. The methylerythritol phosphate (MEP) pathway in the chloroplast is primarily responsible for producing monoterpenoids, diterpenoids, essential photosynthesis pigments (carotenoids and chlorophylls), and plastidial quinones, whereas the mevalonic acid (MVA) pathway in the cytosol is responsible for synthesis of sesquiterpenoids, sterols, and secondary triterpenes. (Leivar et al., 2011; Yang et al., 2012). The synthesis of IPP and its allylic isomer DMAPP is the first step in both the MVA and MEP pathways. 3-Hydroxy-3-methylglutaryl-CoA reductase (HMGR) catalyzes the conversion of one molecule of 3-hydroxy-3-methylglutary-CoA (HMG-CoA) and two molecules of triphosphopyridine nucleotide (NADPH) into MVA (Maurey et al., 1986). Because HMGR activity is highly regulated, HMGR is considered the rate-limiting enzyme in the upstream biosynthetic pathway of hemiterpenes and triterpenes (Heuston et al., 2012). In the MEP pathway, 1-deoxy-D-xylulose5-phosphate synthase (DXS) catalyzes the conversion of pyruvate and D-glyceraldehyde3-phosphate (D-3-P) into 1-deoxy-D-xylulose5-phosphate (DXP), and 1-deoxy-D-xylulose5-phosphate reductoisomerase (DXR) converts DXP into 2-*C*-methyl-D-erythritol4-phosphate (MEP) (Rohmer et al., 1996; Lois et al., 2010). DXS and DXR, which control irreversible processes, are also considered vital enzymes in the upstream biosynthetic pathway of isoprenoids.

In recent years, a large number of cDNAs for HMGR genes have been studied in plants including *Catharanthus roseus, Ginkgo biloba, Corylus avellana, Euphorbia pekinensis, Taxus media, Salvia miltiorrhiza, Arabidopsis thaliana, Aconitum heterophyllum, Panax ginseng, Hevea brasiliensis*, and *Cyanotis arachnoidea* (Maldonado-Mendoza et al., 1992; Liao et al., 2004; Shen et al., 2006; Pan et al., 2009; Cao et al., 2010; Luo et al., 2013; Uthup et al., 2013; Li et al., 2014; Qiu et al., 2014). Plant HMGRs are encoded by a family of genes, and various HMGRs regulate the synthesis of diverse functional MVA metabolites (Suzuki and Muranaka, 2007). For example, HMGR1 in potato is responsible for sterol synthesis induced by mechanical damage and HMGR2 and HMGR3 are responsible for pathogen responses (Choi et al., 1992). In addition, the mutant *hmgr1* of *A. thaliana* has been shown to exhibit plant dwarfing, premature senescence, and male sterility (Suzuki et al., 2005). Analysis of *Arabidopsis* mutant lines has shown that HMGR1 was involved in the biosynthesis of sterols and triterpenoids. Steroids and triterpenoids are involved in cell elongation, senescence, and reproduction in plants, indicating that *HMGR1* plays a considerable role in plant development and cell division. HMGR2 is only expressed in *Arabidopsis* meristem and flower organs (Suzuki et al., 2005). Kim et al. (2014) found that the hmgr1-1 mutant phenotype of *Arabidopsis* could be complemented by overexpression of the PgHMGR1 gene, and that *PgHMGR1* overexpression improved sterol and triterpene production in *Arabidopsis* and ginseng. *PgHMGR1* complemented the phenotypic defect of hmgr1-1, whereas that of *PgHMGR2* did not, indicating that PgHMGR1 is a functional ortholog of AtHMGR1. The overexpression of HMGR from rubber tree (*Hevea brasiliensis*) in tobacco led to a 4- to 8-fold increase in the apparent HMGR activity over the wild-type (WT) control, and phytosterol content was also significantly increased (Schaller et al., 1995). HMGR overexpression in *C. roseus* hairy roots (Ayora-Talavera et al., 2002) caused HMGR activity to increase significantly, and the contents of lycopene, phytosterol, and other terpene secondary metabolites in *C. roseus* were higher than those in the control. In a study on transgenic avocado it was shown that the fruits were much larger than those of the WT, indicating that HMGR plays a significant role in regulating fruit size (Cowan et al., 2001). The effect of SmHMGR2 overexpression on the synthesis of terpenoids in *S. miltiorrhiza* was studied, and both the squalene content in roots and tanshinone content in transgenic *S. miltiorrhiza* were significantly higher than in the control (Dai et al., 2011). Because many terpenoids are involved in the indirect defense of plants (Alborn et al., 2007), the HMGR gene family is a good system for studying plant defense responses. Terpenoids provide a strong defense against, and are eventually toxic to, some insect species (Wu et al., 1997). Statins are effective competitive inhibitors of HMGR and thus can inhibit HMGR activity. Mevinolin was used to study cell cycle progression in synchronized tobacco BY-2 cells; it clearly led to cell cycle arrest, and also induced cell death in a number of cells (Hemmerlin and Bach, 1998). Mevinolin has been shown to have negative effects on the growth, sterol formation, and pigment accumulation of radish seedlings (Bach and Lichtenthaler, 1983). Ginsenoside content was lower in adventitious roots from 4-week-old *P. ginseng* treated with mevinolin for 1 day than in controls, indicating the regulatory role of HMGR in the metabolic fluctuation of ginsenoside (Kim et al., 2014). Previous studies have shown that overexpression of plant *HMGR* can promote an increase of isoprenoids in the metabolic pathway; moreover, inhibition of HMGR activity by enzyme inhibitors significantly reduces isoprenoids and causes plant phenotype changes, indicating that HMGR plays a critical role in isoprenoid synthesis and plant growth and development. Poplar is an adaptable tree species that is widely cultivated in temperate and cold temperate regions throughout the Northern Hemisphere (latitude, 22–70°N), including China, Russia, Canada, the United States, and Europe. Poplar has been used in studies of perennial plant genomes due to the availability of its genome sequence (Tuskan et al., 2006), and genetic engineering has often been used to modify poplars for adaptation to a variety of environments. Transformed, rapidly growing poplar trees overproducing glutathione, a precursor of phytochelatins, have been suggested for the remediation of heavy metal-contaminated soils (Eapen and D’Souza, 2005; Marmiroli et al., 2011). However, in-depth analysis of a *HMGR* gene of *P. trichocarpa*, which codes for the rate-limiting enzyme in cytosolic terpenoid synthesis, has not yet been conducted. Furthermore, its putative effects on terpenoids through differential regulation of the HMGR gene of *P. trichocarpa* remain unclear.

In this study, we cloned a full-length cDNA encoding *HMGR* (XP_002300544.1) from *P*. *trichocarpa*. Additionally, PtHMGR was expressed in *E*. *coli* BL21 (DE3) for purification of the target protein. Functional analyses of the purified target protein were carried out using an *in vitro* enzymatic reaction. Moreover, we investigated the expression pattern of the *PtHMGR* gene in different tissues under abiotic stresses. Furthermore, *PtHMGR* was overexpressed in Nanlin895 (*Populus*× *euramericana* cv. ‘Nanlin895’) poplar. The levels of MEP-, MVA-, downstream-, abscisic acid (ABA)-, gibberellic acid (GA)-, and auxin (IAA)-related transcripts were compared between transgenic and WT poplars. In addition, the measured contents of ABA, GA, α-carotene, β-carotene, and lycopene were significantly higher than those in the control. In both poplars, *PtHMGR* overexpression led to the upregulation of *DXS*, *DXR*, *HDS*, *HDR*, *IDI*, *GPS*, *GPPS,* and *GGPPS* in the MEP pathway, and to α-carotene, β-carotene, and lycopene accumulation. There is literature on this so-called “cross-talk” between the compartmentalized isoprenoid pathways in plants and plant cells. Taken together, these results indicate that the manipulation of *PtHMGR* represents a promising strategy for simultaneous improvement of the levels in poplar of ABA, GA, α-carotene, β-carotene, and lycopene, which are involved in plant growth and responses to different stress factors.

## Materials and Methods

### Plant Cultures, Stresses, and Plasmid Construction

We cultured 3-month-old seedlings of *P*. *trichocarpa* and Nanlin 895 (*Populus× euramericana* cv. ‘Nanlin895’) plants in 1/2 Murashige and Skoog medium (pH 5.8) under 16-h light/8-h dark conditions at 23°C and 74% humidity. Well-grown plants were selected for re-culturing on 1/2 Murashige and Skoog medium containing 200 mM NaCl, 200 µM ABA, and 2 mM hydrogen peroxide (H_2_O_2_). We then collected *P. trichocarpa* seedlings treated with 200 mM NaCl, 200 µM ABA, and 2 mM H_2_O_2_ at 0, 2, 4, 6, 8, 12, 24, and 48 h, and subjected these to 4°C cold stress and 10% PEG_6000_ at 1, 2, 3, 4, 5, 6, and 7 days. Polymerase chain reaction (PCR) was used to amplify the *PtHMGR* gene, and the resulting product was ligated into the PEASY-T3 vector (TransGen Biotech, China) based on TA clone technology. Positive clones were isolated and sequenced (Invitrogen, United States) based on blue-white selection and identified by PCR. The PET-28a, PET-32a, and pSUMO vectors were chosen for cloning and analysis of prokaryotic expression in *E. coli *strains Top10, DH5α, and BL21 (DE3). Total RNA was isolated using the Miniprep kit (Biomiga, United States) according to the manufacturer’s instructions. *Agrobacterium tumefaciens* strain EHA105 was used for infection of leaf discs.

### Gene Isolation and Sequence Analysis

A cDNA library was prepared using poly(A)-enriched mRNA from leaves with the SMART cDNA synthesis kit (TaKaRa Biotechnology Co., Ltd., Japan). The poplar genome database (http://genome.jpi-psf.org/cgi-bin/runAlignment?Db=Poptr1) was used to identify the sequence of the *PtHMGR* gene, and Open Reading Frame (ORF) finder software (http://www.ncbi.nlm.nih.gov/gorf/gorf.html) was used to find ORFs of the *PtHMGR* gene. The conserved sequence of the *HMGR* gene was amplified from the cDNA library through PCR using a set of degenerate primers (**Table S1**: HMGRFor1 and HMGRRev1); the PCR system included 2 µl forward and reverse primers, 2.0 µl cDNA as a template, 5.0 µl 10 × PCR buffer (Mg^2+^), 1 µl 10 mM dNTPs, 0.5 µl rTaq DNA polymerase (TaKaRa), and ddH_2_O added to a constant volume of 50 µl. The PCR reaction was performed as follows: 95°C for 10 min, 35 cycles of 95°C for 1 min, 56°C for 1 min, 72°C for 1.5 min, and 72°C for 10 min. The amplified segment was purified (AXYGEN, United States), ligated to the PEASY-T3 vector (TransGen Biotech), and sequenced. Subsequently, the sequences of the 5’ and 3’ fragments were obtained using gene-specific primers for 5’- and 3’-RACE (**Table S1**: HMGR5’R1, HMGR5’R2 for 5’-RACE and HMGR 3’ F1, HMGR3’F2 for 3’-RACE). The fragments amplified through 5’- and 3’-RACE were inserted into the vector PEASY-T3 and sequenced. The full-length sequence of *PtHMGR* was obtained by aligning these sequences. Finally, the ORF of *PtHMGR* was amplified with gene-specific primers (**Table S1**: ORF-HMGR-F and ORF-HMGR-R), cloned into the vector PEASY-T3, and sequenced.

The nucleotide and amino acid sequences of HMGR obtained from the NCBI database could be identified as conservative fragments based on the alignment generated using the DNAMAN software. MEGA software was used to build a phylogenetic tree using bootstraps of 1,000 replicates. The isoelectric point (pI) and molecular weight of the deduced PtHMGR protein were predicted with the online software pI/Mw (http://www.expasy.org/tools/pi_tool.html). The tertiary structures of PtHMGR and AtHMGR were predicted using the online software tools of the SWISS-MODEL server (http://www.expasy.org/swissmod/SWISS-MODEL.html). The use of the catalytic domain of HMGR only for expression and structure modeling was based on a previously reported X-ray analysis of truncated human HMGR (Istvan et al., 2000).

### Plasmid Construction

Primers for prokaryotic expression were designed according to the sequences of the *PtHMGR* gene and the restriction enzyme sites of the PET-28a, PET-32a, and pSUMO vectors. The *PtHMGR* gene was cloned into these expression vectors between the *Eag*I and *Sal*I sites based on double enzyme digestion (*Eag*I and *Sal*I) and T4 ligation. We selected 306–1,599 base pairs (bp) of *PtHMGR* for heterologous expression of the truncated PtHMGR. The fusion vector PET-28a-truncated-PtHMGR was constructed based on double digestion and T4 ligation.

Recombinant *E. coli* strains BL21 (DE3; bacteria expressing different vectors) were cultivated in 3 ml Luria-Bertani (LB) medium containing 50 µg/ml kanamycin or 30 µg/ml ampicillin. When the optical density (OD_600_) of the bacterial culture reached 0.6–0.8, the remaining solution was induced using 1 mM IPTG and continuously cultivated at 220 rpm for 4 h at 37°C. To determine the effect of temperature on the expression of the target protein, low temperatures (4°C and 10°C) were selected to induce recombinant *E. coli* strains BL21 (DE3). The recombinant solution was cultivated at 37°C until it reached OD_600_ 0.6–0.8. The recombinant solution was then induced using 1 mM IPTG and continuously cultivated at 110 rpm for 72 h at 4°C, or for 48 h at 10°C. Non-induced and induced solutions were analyzed using 12% sodium dodecyl sulfate–polyacrylamide gel electrophoresis (SDS–PAGE).

The target protein was present in the form of insoluble inclusion bodies when the recombinant solution was incubated at 37°C. Denaturation and renaturation processes were carried out. First, the collected inclusion bodies were resuspended three times in resuspension solution 1 (20 mM Tris, 1 mM EDTA, 2 M urea, 1 M NaCl, and 1% Triton X-100, pH 8.0), followed by two resuspensions in resuspension solution 2 (20 mM Tris, 1 mM EDTA, 4 M urea, 1 M NaCl, and 1% Triton X-100, pH 8.0). Then, the inclusion bodies were resuspended once in resuspension solution 3 (20 mM Tris, 1 mM EDTA, 6 M urea, 1 M NaCl, and 1% Triton X-100, pH 8.0). Subsequently, the inclusion bodies were collected and dissolved in resuspension solution 4 (20 mM Tris, 1 mM EDTA, 6 M urea, 1 M NaCl, and 1% Triton X-100, pH 8.0). Finally, the collected denaturation solution was held for 24 h at 4°C and the inclusion bodies were dissolved thoroughly in 8 M urea. In addition, the renaturation process was carried out as follows: the solution described above was added dropwise into renaturation solution (20 mM Tris-HCl, 0.15 M NaCl, and 2-8 M urea, pH 8.0), and then slowly stirred with double gradient dilution. The protein solution was packed into a dialysis bag and dialyzed overnight in dialysis solution (20 mM Tris-HCl and 0.15 NaCl, pH 8.0). The target protein was analyzed using 12% SDS–PAGE.

### Purification of the Target Protein and Western Blotting

Based on the His tag, the fusion protein was applied to a Ni+-NTA-chelating column, then flushed with washing solution (20 mM Tris-HCl, 20 mM imidazole, and 0.15 M NaCl, pH 8.0) until the baseline absorbance was reached. The target protein was flushed with elution solution (20 mM Tris-HCl, 250 mM imidazole, and 0.15 M NaCl, pH 8.0), followed by overnight dialysis at 4°C with dialysis solution (20 mM Tris-HCl and 0.15 M NaCl, pH 8.0). Finally, the protein was lyophilized and fractions were collected and analyzed with 12% SDS–PAGE.

Western blotting was performed with rabbit anti-His polyclonal antibody as the primary antibody and peroxidase-conjugated goat anti-rabbit IgG (Zhongshan Biotechnique) as the secondary antibody.

### *In Vitro* Enzymatic Reaction for Detection of PtHMGR Protein

To assess the activity of the PtHMGR protein, 1 ml of reaction mixture (2.5 mM K_2_HPO_4_, 5 mM KCl, 1 mM EDTA, 5 mM DTT, 1 mg/ml PtHMGR, 3 mM NADPH as a coenzyme and 0.3 mM of HMG-CoA as a substrate, pH 7.2) was incubated at 37°C for 60 min (Bischoff and Rodwell, 1996). The control group was treated similarly, except that the target PtHMGR protein was added to the reaction mixture for identification.

Mass spectrometry (MS) was used to detect the activity of the PtHMGR protein. High-performance liquid chromatography (HPLC; LC20A; Shimadzu, Japan) was adapted using a solvent (0.1% formic acid in methanol) with a column temperature of 30°C, a 0.3 ml/min flow rate, and a 2.1*150 mm XBridge C18 3.5-µm column (Waters, United States). In addition, MS (TSQ 8000 EVO; Thermo, United States) was adapted using a TripleTOF 5600+ m/z (50–1200) instrument (SCIEX, United States) and an information-dependent acquisition (IDA) pattern to collect ions.

### Analysis of Expression Patterns

Various poplar tissues (young and mature leaves, upper and lower stems, petioles, and roots) were treated under different stress conditions (200 mM NaCl, 200 µM ABA, 2 mM H_2_O_2_, 4°C cold stress, and 10% PEG_6000_) to analyze the expression of *PtHMGR* using quantitative reverse-transcription PCR (qRT-PCR). Three plants were used for each replicate. The primers for the *PtActin* gene (accession number: XM-006370951.1), used as an internal reference (Zhang et al., 2013), and the *PtHMGR* gene in qRT-PCR are shown in **Table S1**. The qRT-PCR reactions were performed using PCR SYBR Green Mix (Roche) under the following conditions: initial incubation at 95°C for 5 min, followed by 40 cycles of 30 s at 95°C, 30 s at the annealing temperature at 60°C, and 30 s at 72°C.

### *Agrobacterium*-Mediated Transformation

The ORF the *PtHMGR* gene was cloned into the Gateway entry vector pENTR/D-TOPO (Invitrogen), and then transferred to the destination vector pGWB9 through the LR reaction using LR Clonase II (Invitrogen) according to the manufacturer’s instructions. *PtHMGR* was expressed under the control of the cauliflower mosaic virus (CaMV) 35S promoter. The plasmid was transformed into *A. tumefaciens* strain EHA105. A simplified version of the leaf infection method was used for transformation of Nanlin895 poplar (*Populus× euramericana* cv.), following the specific transformation process described by Movahedi et al. (2015).

After rooting on 1/2 Murashige and Skoog medium, eight lines of putative transformants were selected for confirmation. Genomic DNA was extracted from leaves according to the kit manufacturer’s instructions (Biomiga Miniprep). PCR was used to detect whether the *PtHMGR* gene was inserted into the poplar genome with CaMV35S-F as the forward primer, and the downstream primer of ORF-*PtHMGR* as the reverse primer (**Table S1**: CaMV35S-F and ORF-*PtHMGR*-R). The PCR reaction was carried out under the following conditions: 95°C for 10 min; 35 cycles of 95°C for 1 min, 58°C for 1 min, and 72°C for 1.5 min; and 72°C for 10 min. PCR products were visualized on a 1% agarose gel. Subsequently, qRT-PCR was carried out to reveal the transcript levels of *PtHMGR* in the transformants and WT poplars. The reaction conditions and reaction system for qRT-PCR used for the transformants and WT poplars were carried out as described above.

### Transcript Levels of Genes Related to MVA, MEP, ABA, GA, and IAA

Transcript levels of several genes involved in the MVA and MEP pathways were evaluated in WT and transgenic poplars. In addition, the expression profiles of ABA-, GA-, and IAA-related genes were compared between WT and transgenic poplars. Total RNA was isolated from the leaves of WT and transgenic poplars and subjected to qRT-PCR under the conditions described above. The qRT-PCR primers designed to target MVA-, MEP-, ABA-, GA-, and IAA-related genes are shown in **Table S1**. Three plants were used for each replicate, with three technical repetitions performed for transcription analysis of MVA-, MEP-, ABA-, GA-, and IAA-related genes.

### Quantitative Detection of ABA, GA3, and GA4 Contents

The AB Qtrap6500 mass spectrometer was used in triple four-stage rod-ion hydrazine mode. The ESI-HPLC-MS/MS method was used for the quantitative analysis of phytohormones. Samples were separated using a 1,290 high-performance liquid chromatograph (Agilent, United States) with electrospray ionization as the ion source and scanned in multi-channel detection mode. Three-month-old leaves were chosen for analysis of the contents of ABA, GA3, and GA4. The process of hormone extraction was as follows: 0.5-g fresh plant samples were ground and 10 ml isopropanol/hydrochloric acid mixture was used to dissolve the sample. Next, 20 ml dichloromethane was added to the solution, and the collected solution was centrifuged at 13,000 × *g* for 20 min at 4°C. Then, the organic phase was dried under nitrogen and dissolved in 400 µl methanol containing 0.1% formic acid. Finally, the collected solution was filtered through a 0.22-µm membrane and detected using HPLC-MS/MS. Three independent biological experiments were performed.

The standard solutions were formulated as follows: ABA, GA3, and GA4 at 0.1, 0.2, 0.5, 2, 5, 20, 50, and 200 ng/ml were dissolved in methanol/0.1% formic acid. During plotting of the standard curve, outliers can be excluded. The liquid phase conditions were as follows: a Poroshell 120 SB-C18 column (2.1 × 150, 2.7 m) was used in this study at a column temperature of 30°C. The mobile phase included A:B = (methanol/0.1% formic acid): (water/0.1% formic acid). Elution gradient: 0–1 min, A = 20%; 1–9 min, A = 80%; 9–10 min, A = 80%; 10–10.1 min, A = 20%; 10.1–15 min, A = 20%. The injection volume was 2 µl. MS conditions were as follows: air curtain gas, 15 psi; spray voltage, 4,500 v; atomization pressure, 65 psi; auxiliary pressure, 70 psi; atomization temperature, 400°C.

### Quantitative Determination of α-Carotene, β-Carotene, and Lycopene Contents

First, α-carotene, β-carotene, and lycopene in poplar leaves were extracted according to the following steps: 1.0 g of poplar leaves were ground and 10 ml acetone-petroleum ether (1:1) was used to dissolve the sample, and the collected solution was transferred to a liquid separation funnel and layered statically. Subsequently, the collected solution was evaporated and filtered through a 0.45-µm membrane for HPLC analysis. The Symmetry Shield RP18 reversed-phase chromatographic column (Waters, United States) was used in this study with a column temperature of 30°C. The injection volume was 10 µl. Three independent biological experiments were performed. In addition, the standard curves of α-carotene, β-carotene, and lycopene were generated as described above.

## Results

### Molecular Cloning and Bioinformatics Analysis

In this study, a full-length cDNA of the *HMGR* gene was isolated from *P. trichocarpa*, referred to as *PtHMGR* (XP_002300544.1). The *PtHMGR* ORF is 1,734 bp in length and encodes a peptide of 577 amino acids (**Figure S1**). Its predicted theoretical pI is 6.64, and its predicted theoretical molecular weight is 61.64 kD. Multiple alignment of amino acid sequences revealed that the PtHMGR protein was highly homologous to HMGR in other plant species, with 76.46%, 73.68%, 77.11%, 74.88%, 77.07%, 76.75%, and 76.20% identity with those of *Jatropha curcas* (XP_012087580.1), *Nicotiana tabacum* (XP_016447095.1), *Camellia sinensis* (AHB64333.1), *Nelumbo nucifera* (XP_010270571.1), *Hevea brasiliensis* (XP_021686529.1), *Ricinus communis* (XP_002510732.1), and *Euphorbia lathyris* (AFZ93642.1), respectively (**Figure S2**). The relatively well-conserved domains of the PtHMGR protein were similar to those of other plants. The amino acid sequence of PtHMGR contains two HMG-CoA-binding motifs (EMPVGYIQIP’ and ‘TTEGCLVA) and two NADPH-binding motifs (DAMGMNMV’ and ‘VGTVGGGT) (**Figure S2**).

**Figure 1:**
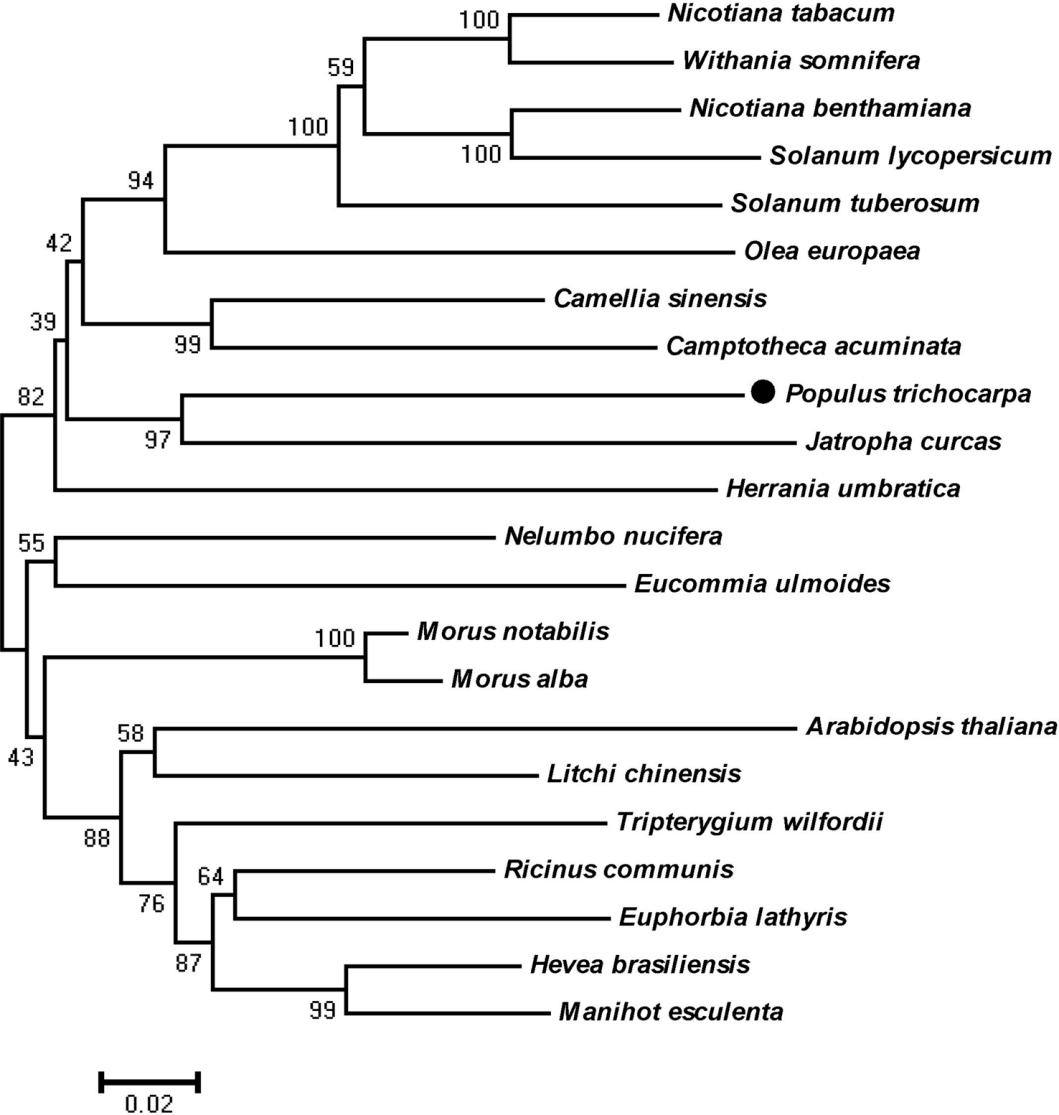
Phylogenetic tree showing relationships between the PtHMGR (XP_002300544.1) amino acid sequence and other identified HMGR amino acid sequences. The tree was constructed using the neighbor-joining (NJ) method in the MEGA 5.1 software and bootstrapped 1,000 times. Bootstrap percentages are indicated at branch points. In all cases, tree topologies obtained using the NJ, minimum evolution, and maximum parsimony methods were identical. Accession numbers of the 3-hydroxy-3-methylglutaryl-CoA reductase (HMGR) sequences obtained from GenBank are as follows: *Arabidopsis thaliana* (NP_177775.2), *Camellia sinensis* (XP_028116621.1), *Camptotheca acuminata* (AAB69727.1), *Eucommia ulmoides* (AAV54051.1), *Euphorbia lathyris* (AFZ93642.1), *Herrania umbratica* (XP_021282837.1), *Hevea brasiliensis* (XP_021686529.1), *Jatropha curcas* (XP_012087580.1), *Litchi chinensis* (ABF56518.2), *Manihot esculenta* (XP_021613442.1), *Morus alba* (AAD03789.1), *Morus notabilis* (XP_010094509.1), *Nelumbo nucifera* (XP_010270571.1), *Nicotiana benthamiana* (BAR94040.1), *Nicotiana tabacum* (XP_016447095.1), *Olea europaea* (AQX83308.1), *Ricinus communis* (XP_002510732.1), *Solanum lycopersicum* (NP_001296119.1), *Solanum tuberosum* (NP_001274940.1), *Tripterygium wilfordii* (AKQ98176.1), and *Withania somnifera* (AOX15271.1).

According to X-ray analysis of truncated human HMGR (Istvan et al., 2000), a protein segment (residues 102–533AA) was chosen. HMGR proteins of other plants were compared to that of *P*. *trichocarpa* through cluster analysis (**Figure 1**). The results clearly showed that PtHMGR had high homology with other amino acid sequences of HMGR proteins, and had the closest evolutionary relationships with other HMGR proteins, such as those from *J*. *curcas* (XP_012087580.1) and *H. brasiliensis* (XP_021686529.1) (**Figure 1**).

### Prokaryotic Expression

**Figure 2:**
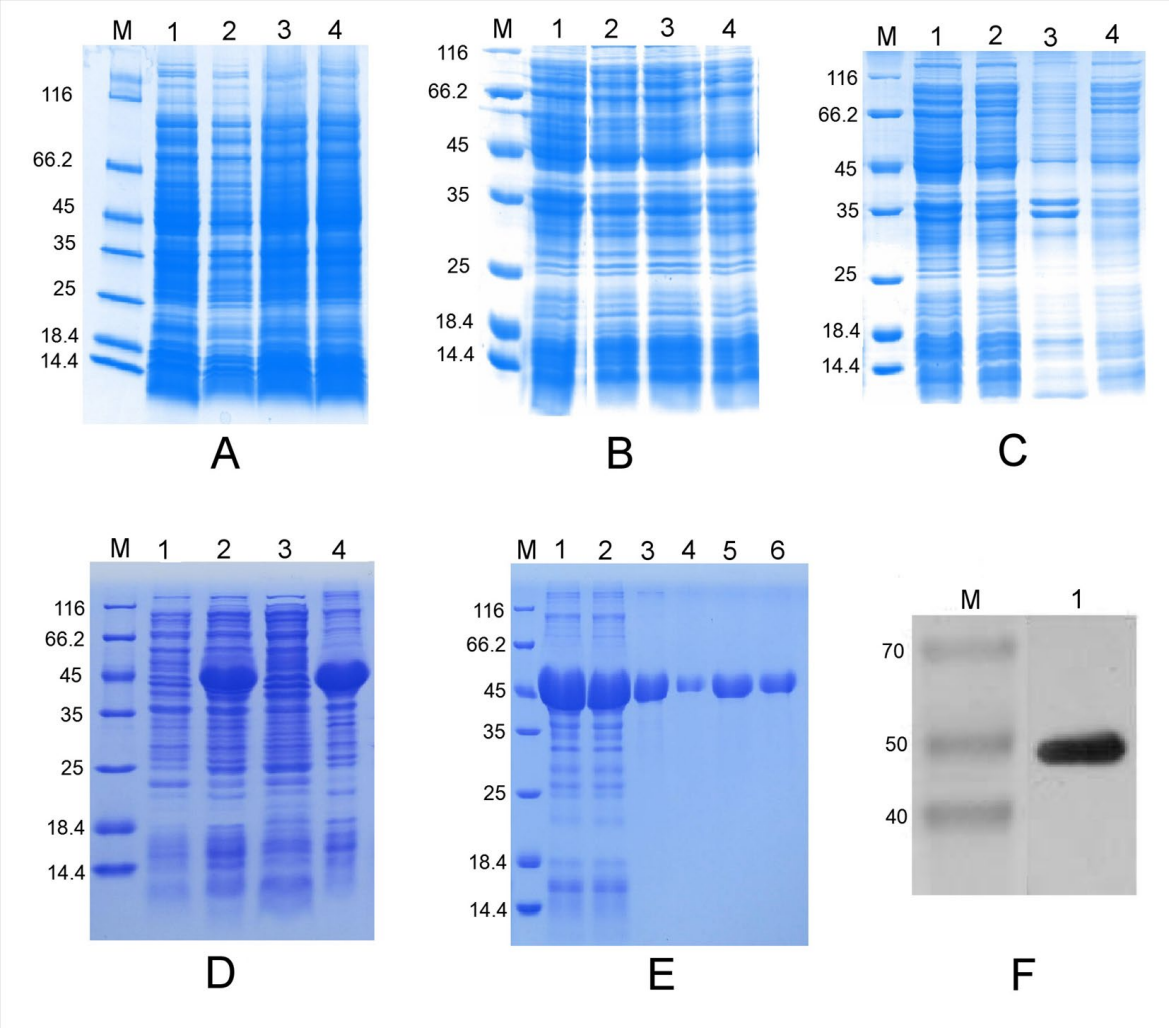
Prokaryotic expression analysis of various expression vectors and purification of the truncated PtHMGR protein. **(A)** Analysis of the expressed fusion protein based on the PET-28a-PtHMGR fusion vector. Lane M: molecular mass marker; lane 1: negative control; lanes 2–4: colonies 1–3, respectively, induced with 1 mM IPTG. **(B)** Analysis of the expressed fusion protein based on the pSUMO-PtHMGR fusion vector. Lane M: molecular mass marker; lane 1: negative control; lanes 2–4: colonies 1–3, respectively, induced with 1 mM IPTG. **(C)** Analysis of the expressed fusion protein based on the PET-32a-PtHMGR fusion vector. Lane M: molecular mass marker; lane 1: negative control; lanes 2–4: colonies 1–3, respectively, induced with 1 mM IPTG. **(D)** Analysis of the expressed truncated PtHMGR protein based on the PET-28a-truncated PtHMGR fusion vector. Lane M: molecular mass marker; lane 1: negative control; lane 2: colony induced with 1 mM IPTG; lane 3: supernatant; lane 4: precipitate. **(E)** Purification of the truncated PtHMGR protein. Lane M: molecular weight marker; lane 1: precipitate after renaturation; lane 2: flow-through; lanes 3–6: eluate. **(F)** Western blot analysis of purified truncated PtHMGR protein using a monoclonal antibody against the His tag. Lane M: molecular weight marker; lane 1: results of western blotting for purified truncated PtHMGR protein.

The ORF of the *PtHMGR* gene was inserted into the prokaryotic expression vectors PET-28a, PET-32a, and pSUMO between the *Eag*I and *Sal*I sites according to analysis of restriction sites (**Figures S3A–C**). IPTG-induced *E*. *coli* BL21 (DE3) revealed no target band in 12% SDS–PAGE analysis (**Figures 2A–C**). The nucleic acid sequence (306–1,599 bp) was amplified by PCR and inserted into the PET-28a plasmid (**Figure S3D**). IPTG-induced bacteria were analyzed using 12% SDS–PAGE to reveal one specific band of the expected 47.33 kDa size (**Figure 2D**). In addition, the truncated PtHMGR protein was detected in the precipitate when the recombinant solution was induced using 1 mM IPTG and incubated at 37°C and considered an inclusion body according to analysis of the supernatant and precipitate (**Figure 2D**). His-tagged proteins were captured using Ni-IDA resin, washed repeatedly in buffer containing 20 mM imidazole, and eluted in buffer containing 250 mM imidazole (**Figure 2E**). Western blots showed that the expression of truncated PtHMGR could be specifically recognized by rabbit antiserum raised against His tag (**Figure 2F**), confirming that the protein expressed by *E. coli* BL21 (DE3) corresponded to PtHMGR.

IPTG-induced *E. coli* BL21 (DE3) were cultivated at 110 rpm for 72 h at 4°C or for 48 h at 10°C. The results of 12% SDS–PAGE showed that the truncated PtHMGR protein was present in both the supernatant and precipitate (**Figures S4A–D**). We then successfully isolated and purified the truncated PtHMGR protein from the supernatant based on the Ni-IDA resin (**Figure S4E**).

### Functional Identification of PtHMGR *In Vitro*

**Figure 3:**
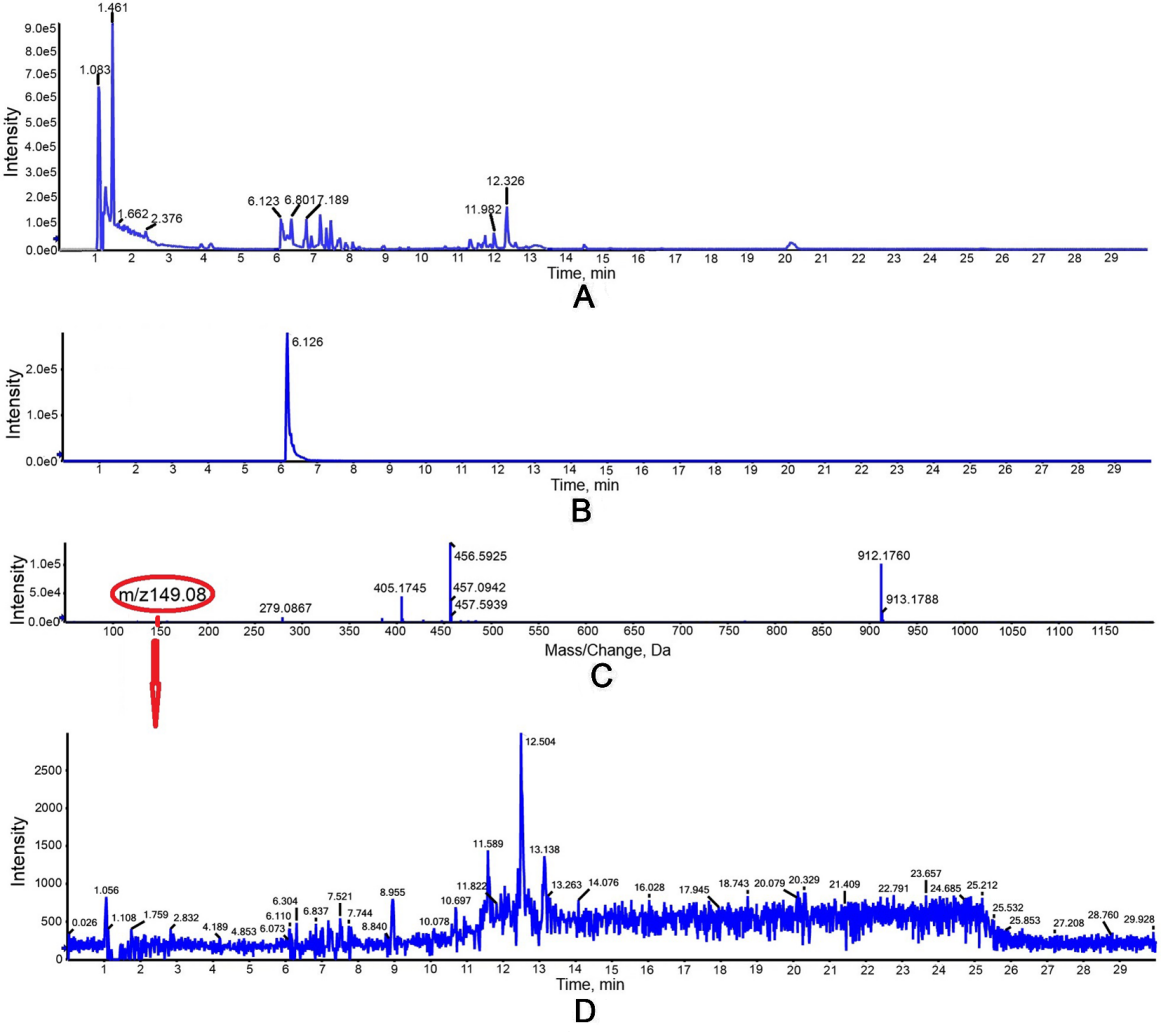
Detection of the negative control group *in vitro* with a 1 ml reaction mixture (2.5 mM K2HPO4, 5 mM KCl, 1 mM EDTA, 5 mM DTT, 3 mM NADPH as a coenzyme, and 0.3 mM of HMG-CoA as a substrate, pH 7.2). **(A)** Total ion chromatogram of reaction products of high-performance liquid chromatography (HPLC). **(B)** Extracted ion chromatography (XIC) analysis. **(C)** Time-of-flight mass spectrometry (TOFMS) analysis. **(D)** XIC analysis of m/z 149.08.

**Figure 4:**
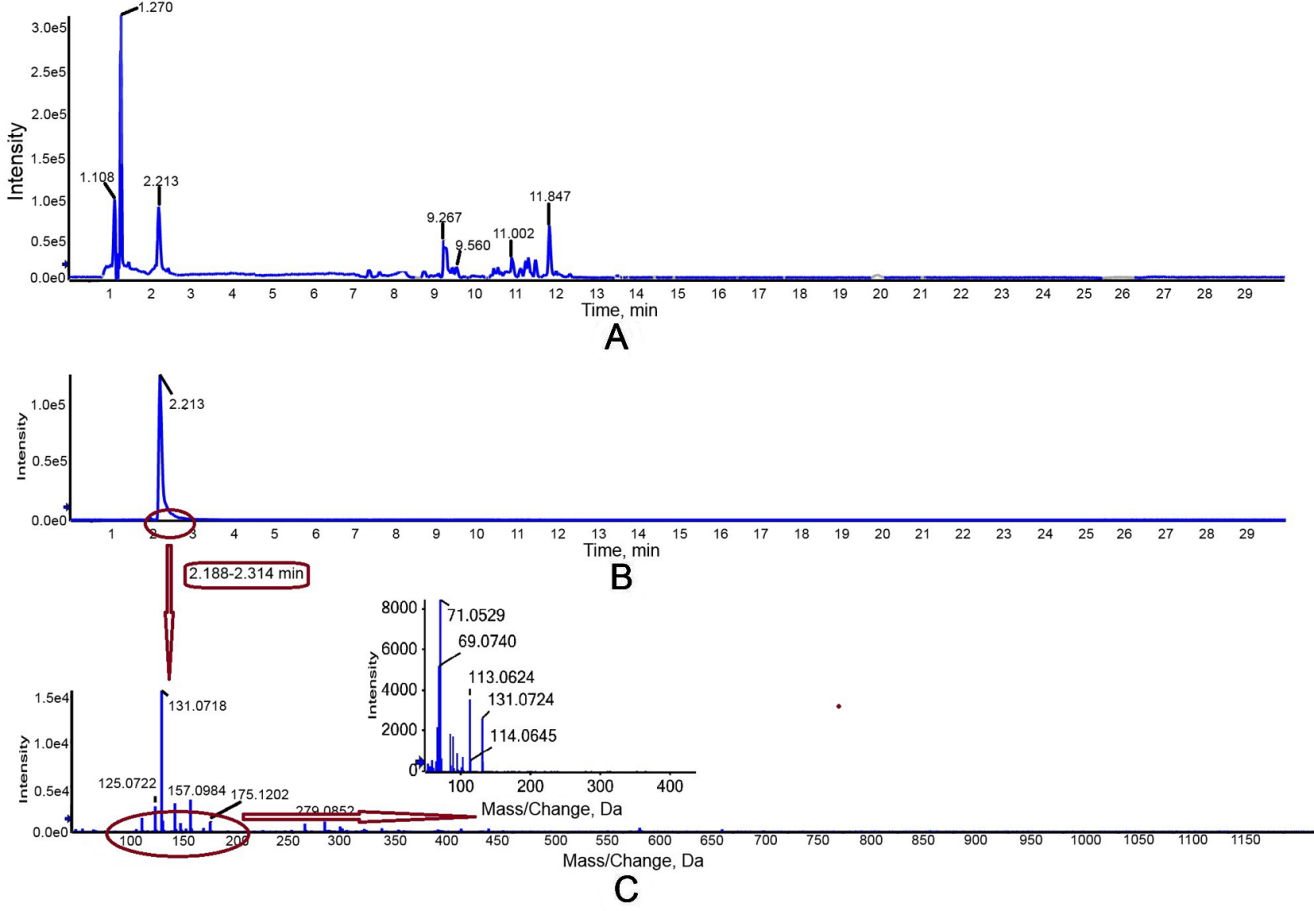
Detection of truncated PtHMGR protein activity *in vitro* with a 1 ml reaction mixture (2.5 mM K_2_HPO_4_, 5 mM KCl, 1 mM EDTA, 5 mM DTT, 1 mg/ml PtHMGR, 3 mM NADPH as a coenzyme, and 0.3 mM of HMG-CoA as a substrate, pH 7.2). **(A)** Total ion chromatogram of reaction products of high-performance liquid chromatography (HPLC). The peak at retention time 2.2 was attributed to target production (MVA). **(B)** Extracted ion chromatography (XIC) analysis. **(C)** Time-of-flight mass spectrometry (TOFMS) analysis. The SCIEX TripleTOF 5600+ m/z value was 131.0710, consistent with MVA.

The activity of purified truncated PtHMGR protein was analyzed using HPLC/MS (**Figures 3** and **4**), and the special peak or mass fragmentation pattern of target production (MVA) was detected at 2.2 min, and the SCIEX TripleTOF 5600+ m/z value was 131.0710 (**Figure 4**). However, for controls, no special peak or MVA was detected at 2.2 min, and the SCIEX TripleTOF 5600+ m/z value differed from 131.0710 (**Figure 3**). Functional evidence was provided by the formation of MVA from HMG-CoA, catalyzed by truncated PtHMGR. The activity of truncated PtHMGR protein in the supernatant was also analyzed using HPLC/MS. The results showed that truncated PtHMGR catalyzed the conversion of HMG-CoA and NADPH into MVA (**Figure S5**); also, in the almost same retention time, we found a higher intensity from activity of PtHMGR in supernatant in comparing with that in inclusion bodies. We speculated that the activity of truncated PtHMGR in the supernatant was orders of magnitude higher than that of truncated PtHMGR in inclusion bodies (**Figure 4** and **Figure S5**).

### Expression of *PtHMGR* in Different Tissues

**Figure 5:**
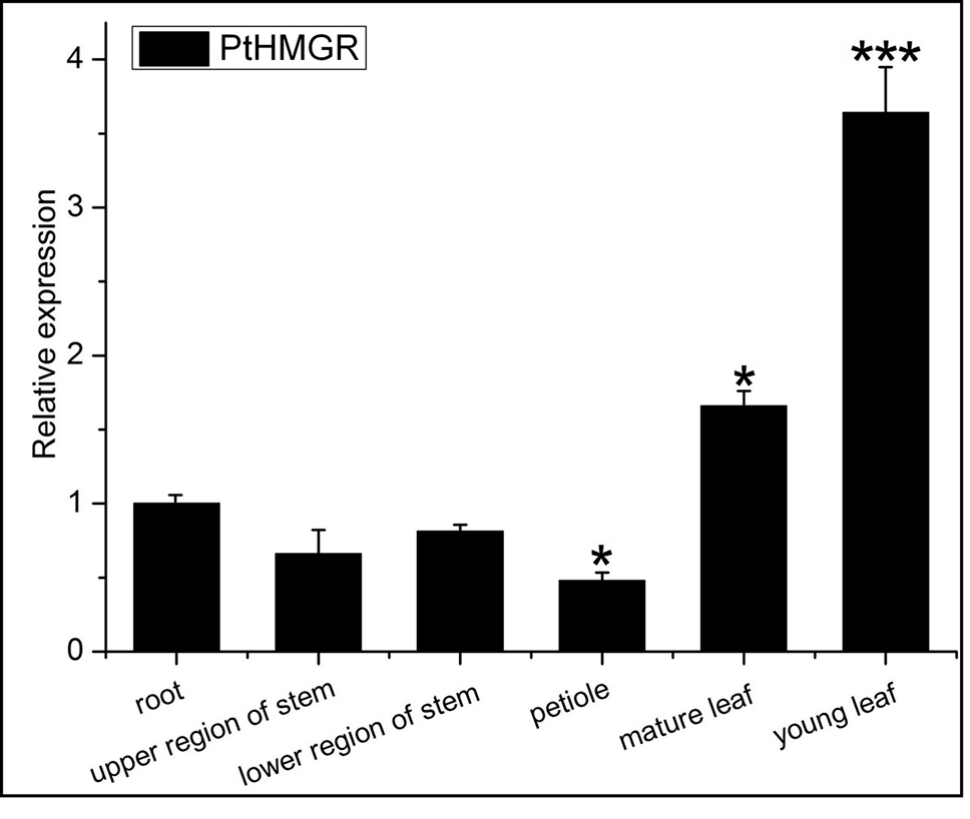
Expression analysis of the *PtHMGR* gene in various tissues. Mean levels (with standard deviation [SD]) in six tissues were analyzed using quantitative reverse-transcription polymerase chain reaction (qRT-PCR). Data presented are 2^–ΔΔCt^ levels calculated relative to a selected tissue (root), which was set to 1, and normalized to the mRNA level of *PtActin*. Three independent biological replicates were analyzed with three technical repeats. Vertical bars represent means ± SD (n = 3). *: significant difference at P < 0.05. ***: significant difference at P < 0.001.

**Figure 6:**
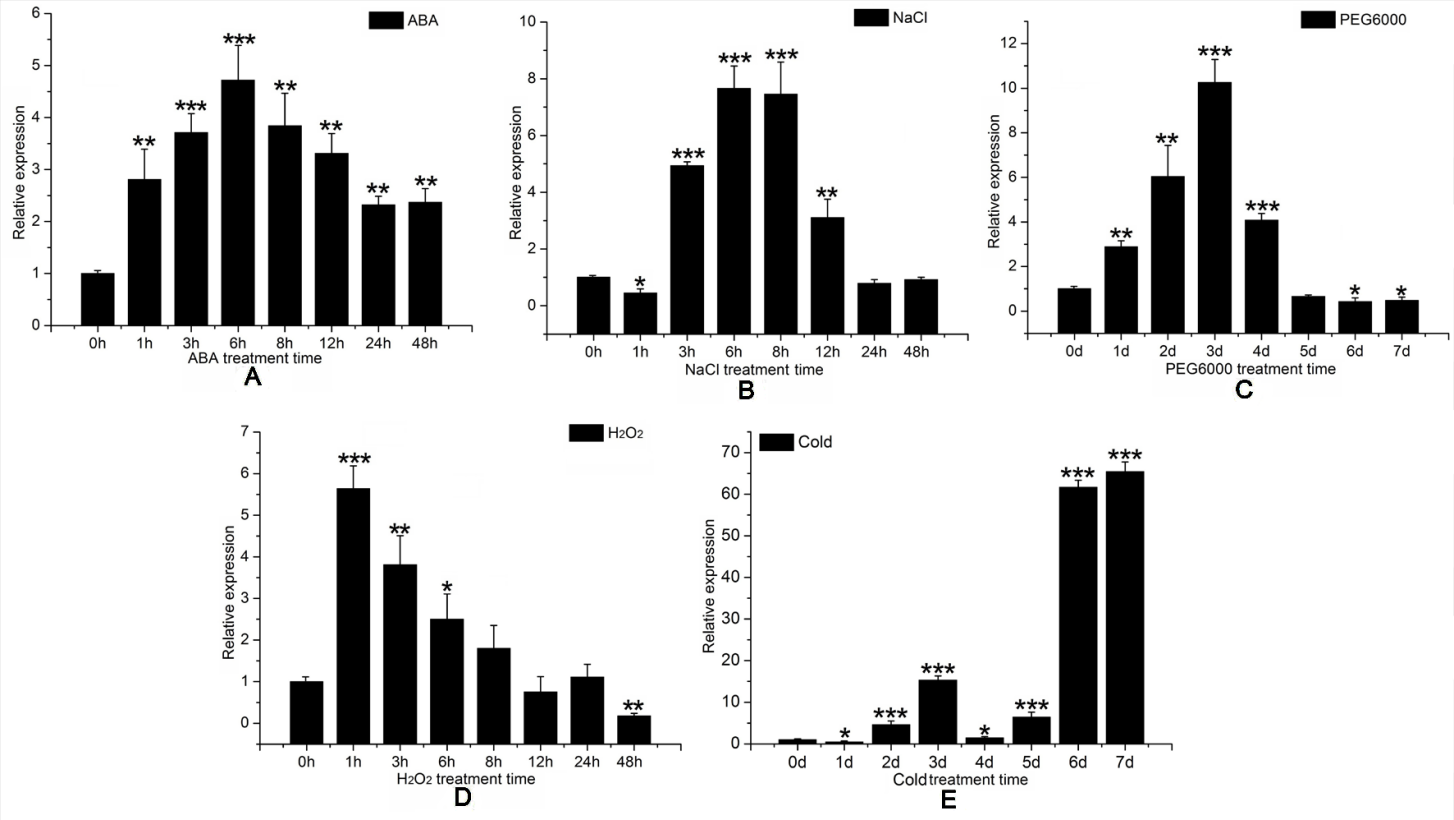
Expression time-course of the *PtHMGR* gene in response to different stress treatments, as determined through quantitative reverse-transcription polymerase chain reaction (qRT-PCR) performed using total RNA extracted from leaves at the indicated times following treatment with 200 μM abscisic acid (ABA) **(A)** 200 mM NaCl, **(B)** 10% PEG_6000_, **(C)** 4°C, and **(D)** 2 mM hydrogen peroxide (H_2_O_2_). **(E)** Relative expression was calculated using *PtActin* as an internal reference. Three independent biological replicates were analyzed with three technical repeats. Vertical bars represent means ± SD (n = 3). *: significant difference at P < 0.05. **: significant difference at P < 0.01. ***: significant difference at P < 0.001.

We then consulted the “Phytozome 12” data bank; after summarizing a number of sequenced poplar genomes, we found six HMGR-like sequences localized to chromosomes 1, 2, 4, 5, 9, and 11. Multiple sequence alignment was performed to identify specific primers (**Table S1**) for *PtHMGR* (Potri.001G457000.1) (**Figure S6**). The qRT-PCR results suggested that *PtHMGR* is expressed in all tissues tested, including mature and young leaves, upper and lower stems, petioles, and roots, with the highest expression seen in leaves, especially young leaves (**Figure 5**).We studied the expression of *PtHMGR* in non-transformed plants treated by exogenous application of ABA, NaCl, PEG_6000_, and H_2_O_2_ as well as cold stress. qRT-PCR showed that the expression of *PtHMGR* in leaves was significantly upregulated under stress conditions. With ABA treatment, the expression of *PtHMGR* was significantly upregulated from 1 to 48 h, with a peak at 6 h post-treatment (**Figure 6A**). Treatment with 200 mM NaCl caused significant accumulation of *PtHMGR* mRNA from 3 to 12 h of salt stress (**Figure 6B**). The expression of *PtHMGR* was enhanced with 1 to 4 days of 10% PEG_6000_ treatment, then the transcript level of PtHMGR declined from 5 days (**Figure 6C**). In addition, treatment with 2 mM H_2_O_2_ caused significant accumulation of *PtHMGR* mRNA from 1 to 6 h (**Figure 6D**). Treatment with cold stress resulted in an increase in the transcript level of *PtHMGR* of nearly 60-fold at 7 days (**Figure 6E**). These data suggest that *PtHMGR* may play a specific role in the regulation of pathways induced by abiotic stress in poplar.

### Characterization of Transgenic Nanlin895 Lines

To evaluate the functional role of the *PtHMGR* gene in transgenic poplars, the PGWB9-*PtHMGR* vector was introduced into WT poplars *via Agrobacterium*-mediated transformation (**Figure S7**). Eight independent T0 transgenic poplars were obtained through screening of regenerated kanamycin-resistant poplar plants. Then, T1 regeneration plants were obtained through subculturing. Genomic PCR analysis with specific primers (CaMV35S-F as the forward primer and ORF-*PtHMGR*-R as the reverse primer) showed that eight of the T1 plants had the expected band (**Figure S8A**). Subsequently, the expression levels of *PtHMGR* in various T2 transgenic poplar lines were examined. The relative expression levels of *PtHMGR* increased significantly (**Figure S8B**). Genomic PCR and analysis of *PtHMGR* expression levels in transgenic poplar lines revealed that the *PtHMGR* gene was integrated into the genome and stably expressed in plant cells.

### Overexpression of *PtHMGR* in Transgenic Poplars Significantly Regulates Downstream Genes Involved in the MVA and MEP Pathways

**Figure 7:**
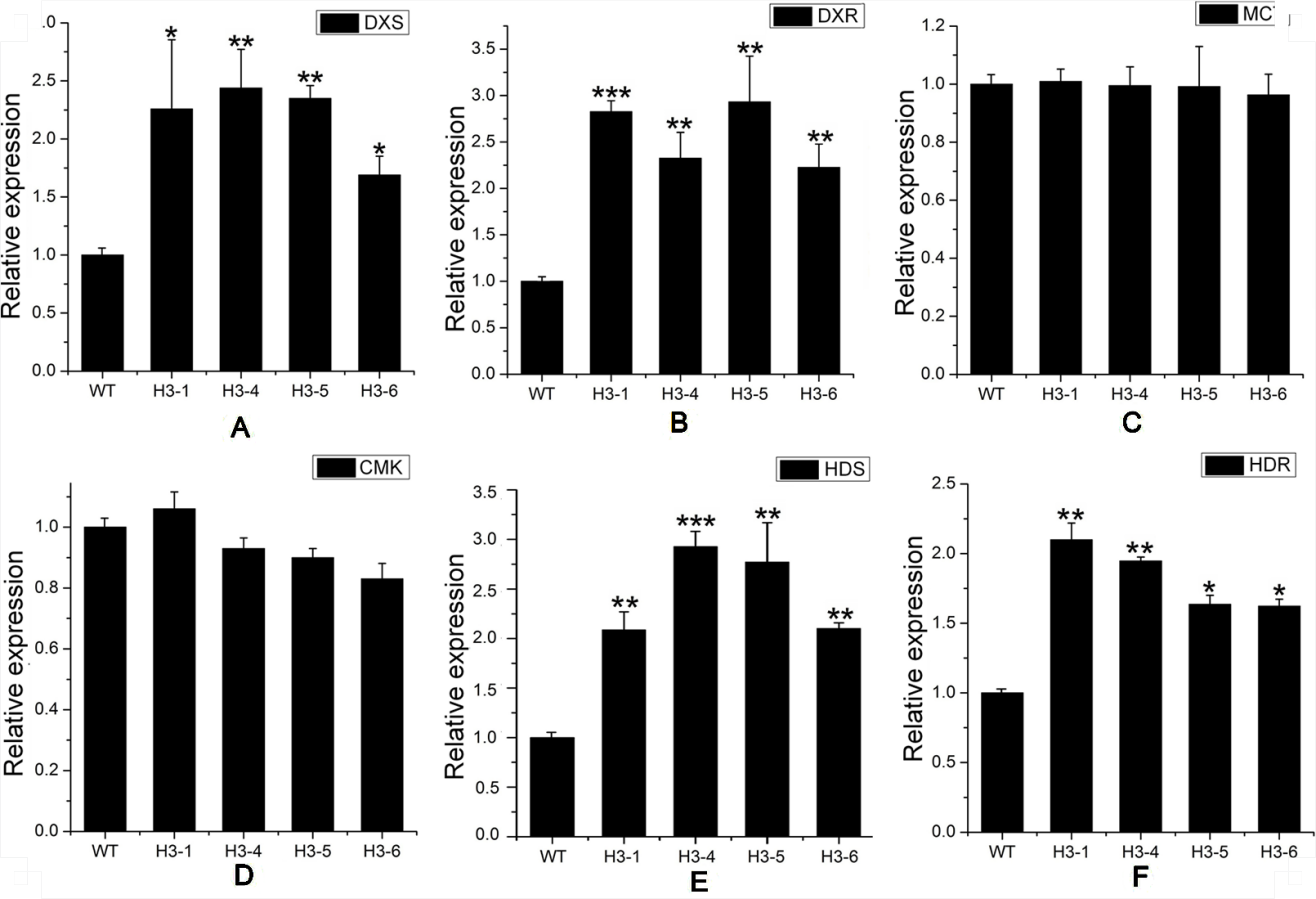
Transcript levels of methylerythritol phosphate (MEP)-related genes in transgenic and wild-type (WT) poplars. Transcript levels of MEP-related genes including **(A)** *DXS* (1-deoxy-D-xylulose5-phosphate synthase), **(B)** *DXR* (1-deoxy-D-xylulose5-phosphate reductoisomerase), **(C)** *MCT* (MEP cytidylyltransferase), **(D)** *CMK* (4-diphosphocytidyl-2-*C*-methyl-D-erythritol kinase), **(E)** *HDS* (1-hydroxy-2-methyl-2-(*E*)-butenyl4-diphosphate synthase), and **(F)** *HDR* (1-hydroxy-2-methyl-2-(*E*)-butenyl4-diphosphate reductase) in transgenic lines and WT. Relative expression was calculated using *PtActin* as an internal reference. Three independent biological replicates were analyzed with three technical repeats. Vertical bars represent means ± SD (n = 3). *: significant difference at P < 0.05. **: significant difference at P < 0.01. ***: significant difference at P < 0.001.

**Figure 8:**
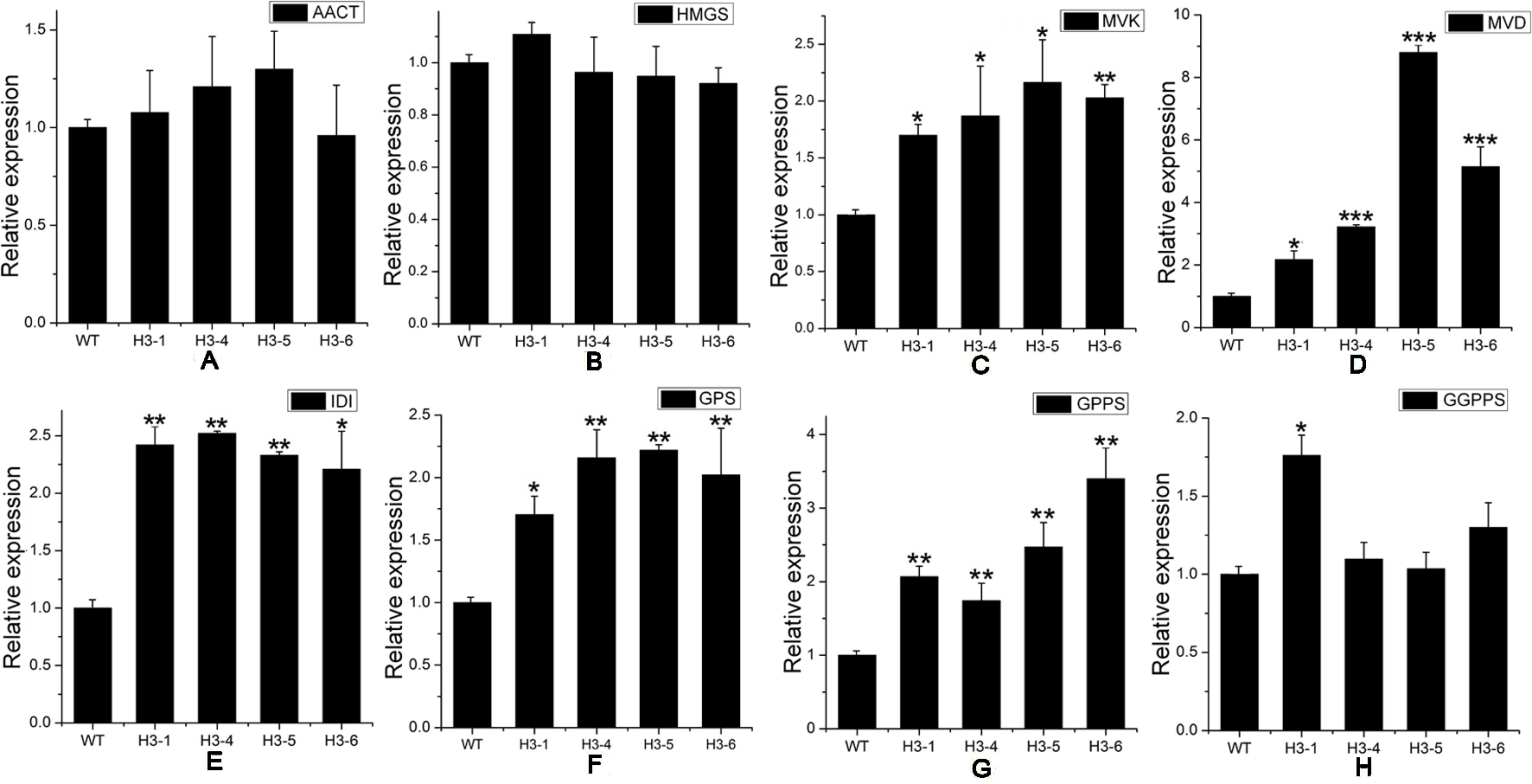
Transcript levels of mevalonic acid (MVA)-, and downstream-related genes in transgenic and wild-type (WT) poplars. Transcript levels of MVA-related genes including **(A)** *AACT* (acetoacetyl CoA thiolase), **(B)** *HMGS* (3-hydroxy-3-methylglutaryl-CoA synthase), **(C)** *MVK* (mevalonate kinase), and **(D)** *MVD* (mevalonate5-diphosphate decarboxylase) in transgenic lines and WT. Transcript levels of downstream-related genes including **(E)** *IDI* (isopentenyl diphosphate isomerase), **(F)** *GPS* (geranyl diphosphate), **(G)** *GPPS* (geranyl diphosphate synthase), and **(H)** *GGPPS* (geranyl geranyl diphosphate synthase) in transgenic lines and WT. Relative expression was calculated using *PtActin* as an internal reference. Three independent biological replicates were analyzed with three technical repeats. Vertical bars represent means ± SD (n = 3). *: significant difference at P < 0.05. **: significant difference at P < 0.01. ***: significant difference at P < 0.001.

As a rate-limiting enzyme in the MVA pathway, HMGR may influence the transcript levels of genes throughout the pathway. Transcript levels of MEP pathway-related genes, including *DXS* (**Figure 7A**), *DXR* (**Figure 7B**), *HDS* (1-hydroxy-2-methyl-2-(*E*)-butenyl4-diphosphate synthase) (**Figure 7E**), and *HDR* (1-hydroxy-2-methyl-2-(*E*)-butenyl4-diphosphate reductase) (**Figure 7F**) were significantly elevated in transgenic lines, while the transcript levels of *MCT* (MEP cytidylyltransferase) (**Figure 7C**) and *CMK* (4-diphosphocytidyl-2-*C*-methyl-D-erythritol kinase) (**Figure 7D**) showed no significant changes between transgenic poplar lines and WT poplars. Among MVA pathway-related genes, there was no significant difference in the expression patterns of *AACT* (acetoacetyl CoA thiolase) (**Figure 8A**) and *HMGS* (3-hydroxy-3-methylglutaryl-CoA synthase) (**Figure 8B**), which are *HMGR* upstream genes, between transgenic lines and WT plants. Transcript levels of *MVK* (mevalonate kinase) and *MVD* (mevalonate5-diphosphate decarboxylase), which are *HMGR* downstream genes, exhibited significant changes, with a greater change in *MVD* expression than that of *MVK* (**Figures 8C, D**). Among downstream-related genes, the transcript expression levels of *IDI* (IPP isomerase) (**Figure 8E**), *GPS* (geranyl diphosphate) (**Figure 8F**), and *GPPS* (geranyl diphosphate synthase) (**Figure 8G**) were strongly increased in transgenic lines. Different transgenic strains showed different changes in the expression level of *GGPPS* (geranyl geranyl diphosphate synthase); expression of *GGPPS* was enhanced significantly in the H3-1 line, while there was a slight increase in the H3-4, H3-5, and H3-6 lines when compared to WT plants (**Figure 8H**).

### Increased Expression of Genes Related to ABA and GA and Altered Expression of Genes Related to IAA Between WT and Transgenic Poplars

**Figure 9:**
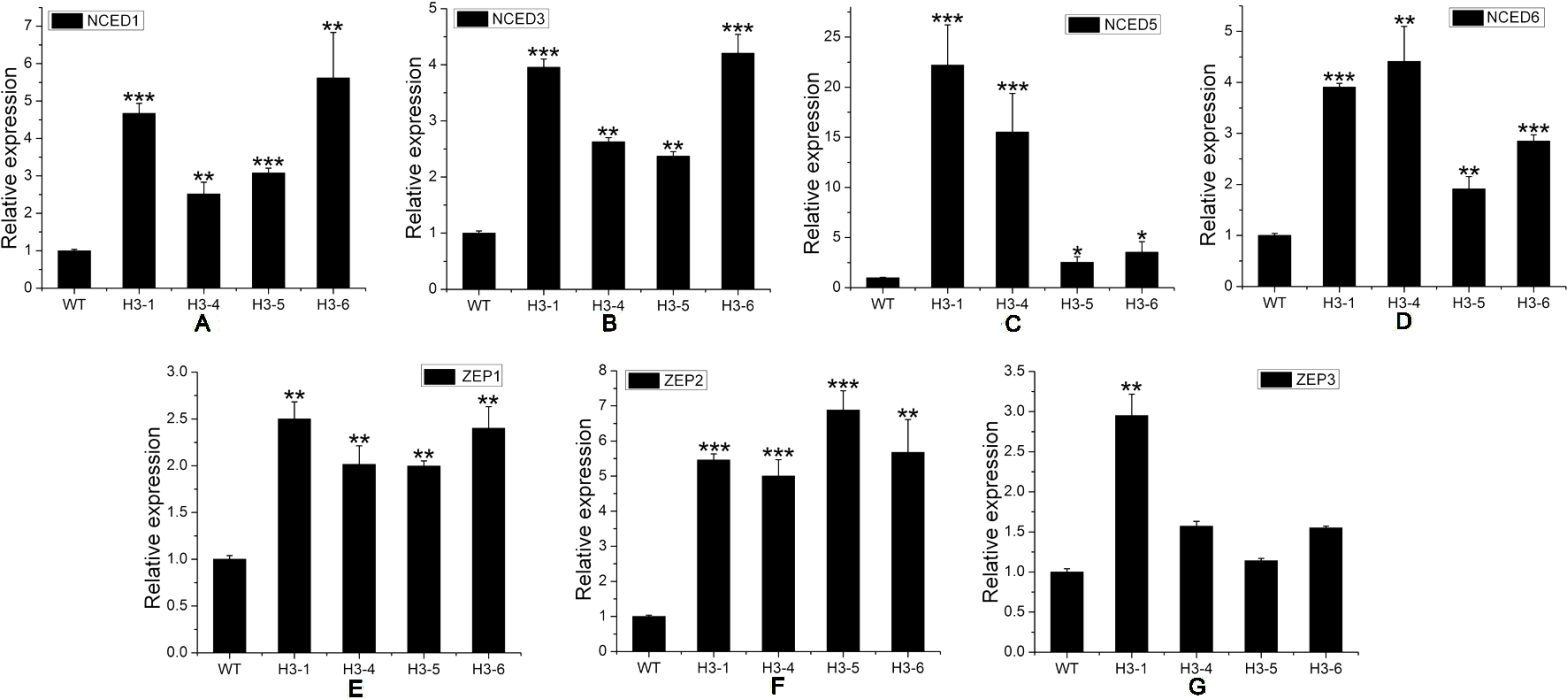
Transcript levels of abscisic acid (ABA)-related genes in transgenic and wild-type (WT) poplars. Transcript levels of ABA-related genes including **(A)** *NCED1*, **(B)** *NCED3*, **(C)** *NCED5*, **(D)** *NCED6*, **(E)** *ZEP1*, **(F)** *ZEP2*, and **(G)** *ZEP3* in transgenic lines and WT poplars. Relative expression was calculated using *PtActin* as an internal reference. Three independent biological replicates were analyzed with three technical repeats. Vertical bars represent means ± SD (n = 3). *: significant difference at P < 0.05. **: significant difference at P < 0.01. ***: significant difference at P < 0.001.

**Figure 10:**
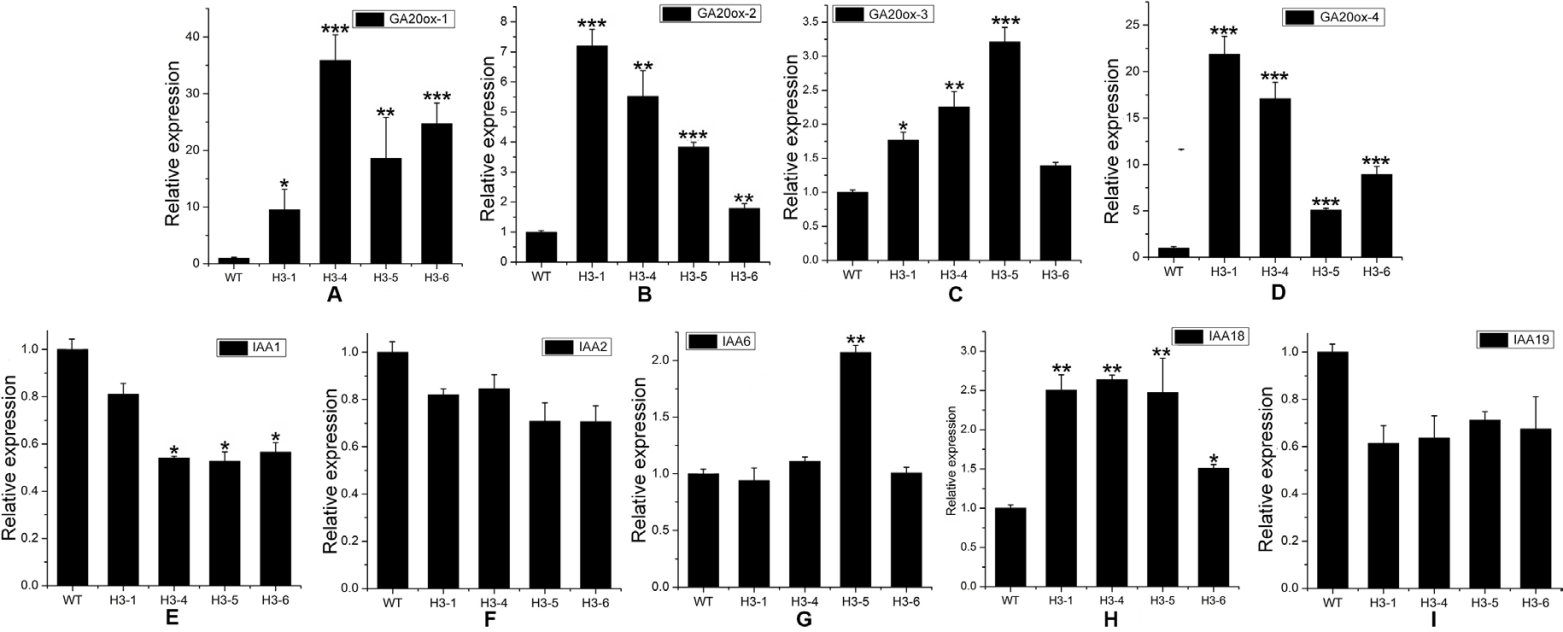
Transcript levels of gibberellic acid (GA)-, and auxin (IAA)-related genes in transgenic and wild-type (WT) poplars. Transcript levels of GA-related genes including **(A)** *GA20_OX_-1*, **(B)** *GA20_OX_-2*, **(C)** *GA20_OX_-3*, and **(D)** *GA20_OX_-4* in transgenic lines and WT. Transcript levels of GA-related genes including** (E)** *IAA1*, **(F)** *IAA2*, **(G)** *IAA6*, **(H)** *IAA18*, and **(I)** *IAA19* in transgenic lines and WT. Relative expression was calculated using *PtActin* as an internal reference. Three independent biological replicates were analyzed with three technical repeats. Vertical bars represent means ± SD (n = 3). *: significant difference at P < 0.05. **: significant difference at P < 0.01. ***: significant difference at P < 0.001.

ABA, a sesquiterpene compound biosynthesized from a C40 carotenoid (Finkelstein, 2013) (**Figure S9A**), plays a variety of roles in plant growth, regeneration, and stress response processes. GA, a diterpene compound, is a widespread plant hormone (Ozga et al., 2009; Reinecke et al., 2013) (**Figure S9B**). ABA-related genes include the *NCED* (9-cis-epoxycarotenoid dioxygenase) and *ZEP* (zeaxanthin epoxidase) families. Our qRT-PCR results indicated that transcript levels of *NCED1*, *NCED3*, *NCED5*, *NCED6*, *ZEP1*, and *ZEP2* were significantly enhanced in transgenic lines (**Figures 9A–F**), and the expression level of *ZEP3* was enhanced significantly in the H3-1 line, while small increases were observed in the expression level of *ZEP2* in the H3-4, H3-5, and H3-6 lines (**Figure 9G**). However, the expression levels of GA-related genes showed significant differences between the transgenic lines and WT poplars (**Figures 10A–D**).

Although IAA is not a terpenoid compound (Mashiguchi et al., 2011) (**Figure S9C**), the transcript levels of IAA-related genes exhibited changes, as shown by the qRT-PCR results. In this study, the genes *IAA1* (auxin response factor 1), *IAA2*, *IAA6*, *IAA18*, and *IAA19* were chosen for analysis of changes between transgenic lines and WT poplars. The expression level of *IAA1* decreased significantly in the H3-4, H3-5, and H3-6 lines, but there was only a slight reduction in the transcript level of *IAA1* in the H3-1 line (**Figure 10E**). For *IAA2*, the changes in all tested transgenic lines were similar, and *IAA2* expression levels decreased slightly when comparing transgenic lines to WT poplars (**Figure 10F**). For *IAA6*, the results showed that there was no significant difference in expression levels between the H3-1, H3-4, and H3-6 transgenic lines and WT poplars, but the transcript levels of *IAA6* exhibited significant differences (**Figure 10G**). The expression of *IAA18* was significantly increased in the transgenic lines (**Figure 10H**), whereas *IAA19* was significantly decreased (**Figure 10I**).

### Overexpression of *PtHMGR* in Transgenic Poplar Increases the Contents of ABA and GA3

**Figure 11:**
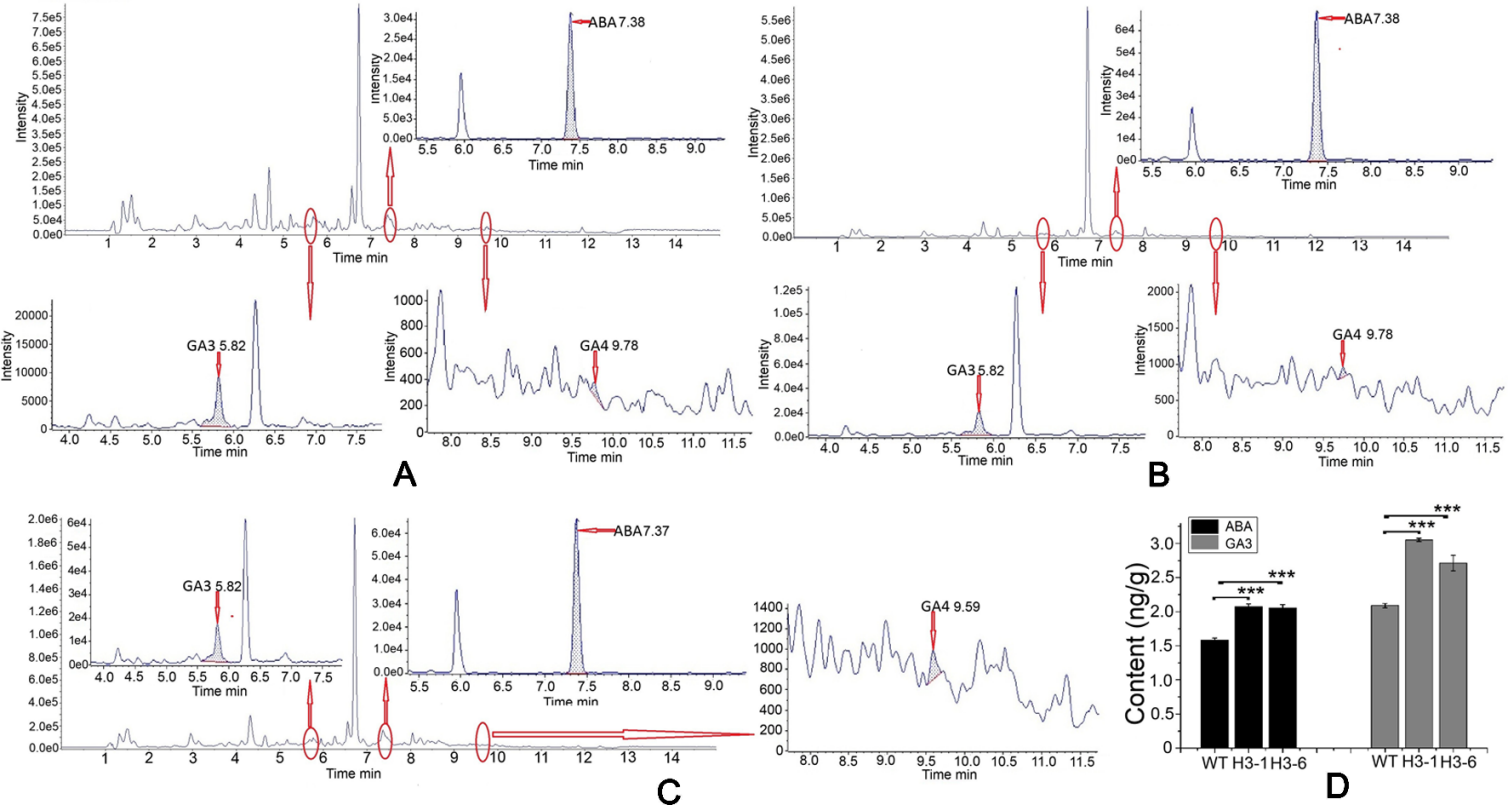
Analysis of abscisic acid (ABA), GA3, and GA4 contents in the transgenic lines and WT poplars using high-performance liquid chromatography (HPLC)–tandem mass spectrometry (MS/MS). **(A)** HPLC–MS/MS chromatogram of WT raw data and analysis of ABA, GA3, and GA4 contents in wild-type (WT). **(B)** HPLC–MS/MS chromatogram of transgenic H3-1 raw data and analysis of ABA, GA3, and GA4 content in the H3-1 line. **(C)** HPLC–MS/MS chromatogram of transgenic H3-6 raw data and analysis of ABA, GA3, and GA4 content in H3-6. **(D)** Analysis of the difference in ABA and GA3 content between transgenic lines and WT poplars. Three independent biological replicates were analyzed with three technical repeats. Vertical bars represent means ± SD (n = 3). ***: significant difference at P < 0.001.

In the ABA standard, 263.1/153.0 and 263.1/204.2 fragments were detected. Of these, 263.1/153.0 fragments had the higher response value, stable and reproducible results, and less interference from impurities. The 263.1/204.2 fragment ions were used as qualitative ions (**Figure S10**). The same method was adapted to identify the GA3 and GA4 standards. For the GA3 standard, the 345.2/239.2 fragment was selected as the quantitative ion, and the 345.2/143.0 fragment was used as the qualitative ion (**Figure S11**). For the GA4 standard product, the 331.4/243.2 fragment ion was selected as the quantitative ion, and the 331.4/213.1 fragment ion was selected as the qualitative ion (**Figure S12**). Identification of ABA, GA3, and GA4 is shown in **Figure 11**. The ABA content in transgenic lines was elevated by 22–36% compared to the control, and the mass of ABA in transgenic lines was greater than 2 ng/g. There was a significant difference in ABA contents between the transgenic lines and WT poplars (**Figures 11A–D**). In addition, the selected transgenic lines produced total GA3 levels 1.25- to 1.5-fold higher than those of the controls. The highest total GA3 levels were detected in the H3-1 line, and the maximum GA3 content in transgenic poplar was 3.08 ng/g (**Figures 11A–D**). By contrast, the GA4 content in transgenic lines and WT poplars was apparently below the detection limit (**Figures 11A–C**).

### Overexpression of *PtHMGR* in Transgenic Poplar Enhances the Contents of α-Carotene, β-Carotene, and Lycopene

**Figure 12:**
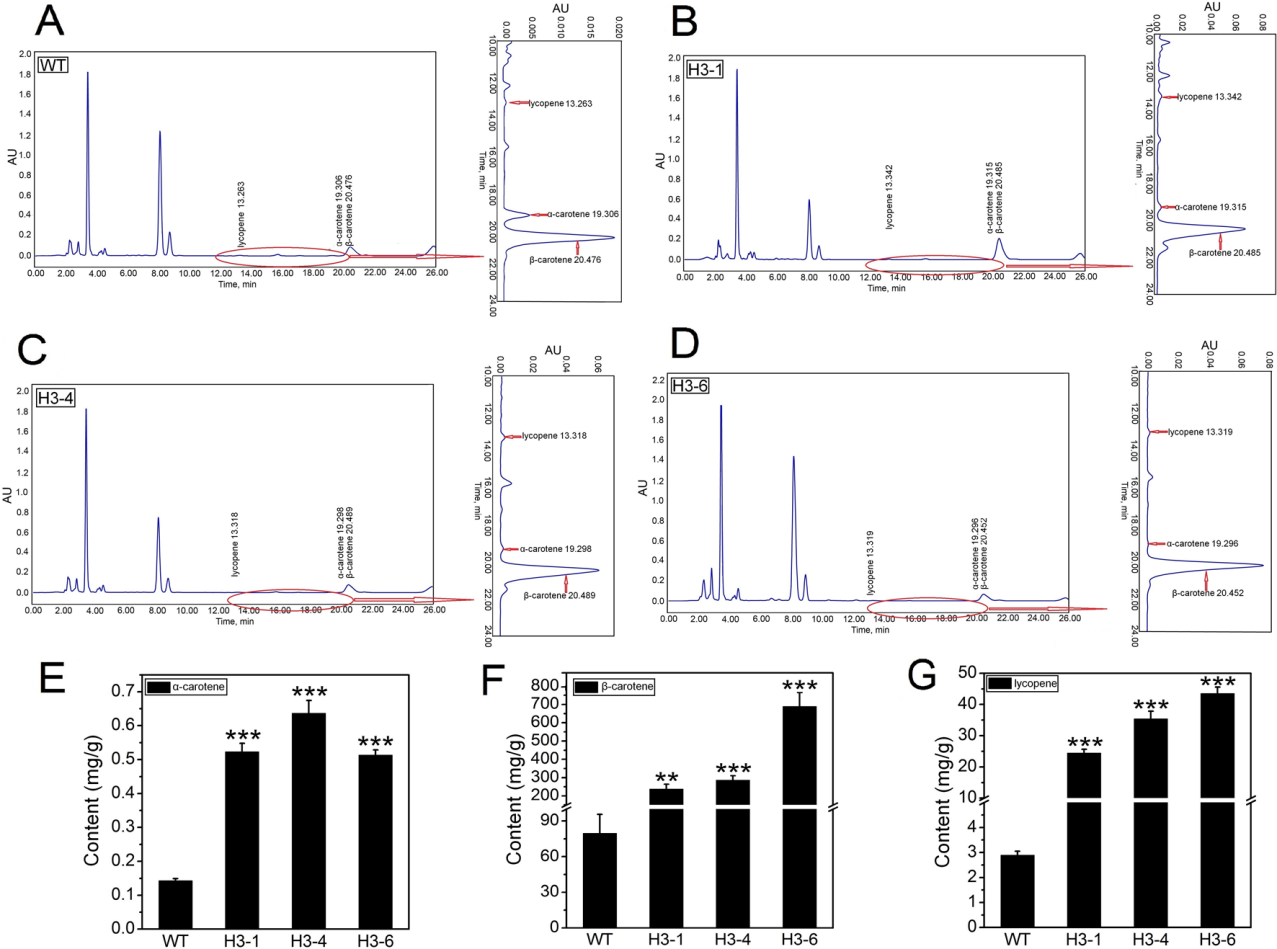
Analysis of α-carotene, β-carotene, and lycopene content in the transgenic lines and WT poplars using HPLC. **(A)** High-performance liquid chromatography (HPLC) chromatogram of α-carotene, β-carotene, and lycopene content in WT. **(B)** HPLC chromatogram of α-carotene, β-carotene, and lycopene content in transgenic H3-1. **(C)** HPLC chromatogram of α-carotene, β-carotene, and lycopene content in transgenic H3-4. **(D)** HPLC chromatogram of α-carotene, β-carotene, and lycopene content in transgenic H3-6. **(E)** Analysis of the difference in α-carotene content between the transgenic lines and WT poplars. **(F)** Analysis of the difference in β-carotene content between the transgenic lines and WT poplars. **(G)** Analysis of the difference in lycopene content between the transgenic lines and WT poplars. Three independent biological replicates were analyzed with three technical repeats. Vertical bars represent means ± SD (n = 3). **: significant difference at p<0.01. ***: significant difference at P < 0.001.

The HPLC method was calibrated according to standard curves of α-carotene, β-carotene, and lycopene (**Figure S13**). To analyze the effect of overexpression of *PtHMGR* on α-carotene, β-carotene, and lycopene biosynthesis, extracts from 6-month-old cultured poplars were analyzed using HPLC (**Figures 12A–D**). The selected transgenic lines produced total α-carotene levels 3.5- to 4.8-fold higher than WT poplars, and the highest content of total α-carotene of ∼0.65 mg/g was detected in the H3-4 line (**Figure 12E**). Identification of β-carotene is shown in **Figure 12F**. β-carotene increased 6.4- to 11.8-fold compared to the control, and the highest β-carotene accumulation was in the H3-6 line when the selected transgenic lines were compared (**Figure 12F**). In addition, the lycopene content of these lines increased significantly by 20- to 40-fold compared to the control levels, and the greatest total lycopene content was detected in line H3-6 (**Figure 12G**).

## Discussion

In the MVA pathway, HMGR catalyzes the conversion of HMG-CoA and NADPH into MVA (Maurey et al., 1986). In previous studies of HMGR activity, color complementation assays have been commonly used to confirm the functions of HMGR enzymes, as *E. coli* with foreign carotenogenic gene clusters introduced can produce and accumulate carotenoid pigments (Matthews and Wurtzel, 2000; Akhtar et al., 2013). Our HPLC/MS analyses demonstrated that PtHMGR synthesizes MVA from HMG-CoA and NADPH, resulting in increased terpenoid content.

Some *HMGR* genes are constitutively expressed and may be involved in regulating basic physiological metabolism. For example, the expression level of the *TmHMGR* gene in *Taxux media* was generally consistent among leaves, roots, and stems (Liao et al., 2004). The transcript level of *EuHMGR* in *Eucommia ulmoides* was higher in stems and leaves, but lower in roots (Jiang et al., 2006). The expression pattern of *SmHMGR2* in *S. miltiorrhiza* was highest in leaves, followed by stems and roots (Dai et al., 2011). In this study, transcript levels of *PtHMGR* in different tissues were analyzed by qRT-PCR; the results indicated that *PtHMGR* expression was highest in young leaves, followed by mature leaves and roots, and lowest in petioles. In addition, exogenous SA, MeJA, and other hormone treatments could induce upregulation of *HMGR* gene expression in plants, such as *C. avellana CgHMGR* (Wang et al., 2007) and *SmHMGR* (Dai et al., 2011), indicating that phytohormones play a significant role in terpenoid metabolism. In this study, we simulated different stress conditions, using 200 mM NaCl, 10% PEG_6000_, and 200 µM ABA as salt, drought, and hormone stress treatments, respectively. The results indicated that *PtHMGR* can be activated in response to stress conditions and may play significant roles in the regulation of terpenoid biosynthesis, growth, and development in poplars.

The HMGR protein determines the direction of carbon flow during the metabolism of terpenoids, so increasing the expression level of *HMGR* may enhance the biosynthesis of terpenoids in plants. HMGR is not only regulated at the gene level, but also by covalent modification (Goldstein and Brown, 1990; Hemmerlin, 2013). In recent years, interest has grown in the regulation of key enzymes involved in the biosynthesis of secondary metabolites. Many studies have shown that overexpressing *HMGR* genes in plants can increase terpenoid contents. For example, the *AtHMGR* gene of *A. thaliana* was overexpressed in tomato, and phytosterol content in tomato increased 2.4-fold (Enfissi et al., 2010). Artemisinin content in *A. annua* increased by 17.4% with co-transfection of *HMGR* and *FPS* genes (Lin et al., 2011). Overexpression of the *HMGR* gene in transgenic *Ganoderma lucidum* led to a 2-fold increase in ganoderic acid content (Xu et al., 2012). Overexpression of *HMGR* in *Parthenium argentatum* contributed to an increase in natural rubber content (Dong et al., 2013). Previous studies have demonstrated that the MEP pathway is mainly responsible for producing monoterpenoids, diterpenoids, and tetraterpenes, while the MVA pathway is responsible for synthesizing sesquiterpenoids and triterpenes (Morris et al., 2011; Yang et al., 2012; Liao et al., 2014). In this study, transgenic lines exhibited increased amounts of ABA synthesized from an indirect pathway through the cleavage of a C40 carotenoid precursor, whereas contents of GA, a diterpenoid, and α-carotene, β-carotene, and lycopene increased significantly in transgenic lines. These results show that overexpression of *PtHMGR* not only increased the content of sesquiterpenes in the MEP pathway but also those of diterpenoids and tetraterpenoids in the MVA pathway. In this study, we demonstrated that manipulation of PtHMGR from the MVA pathway in poplar led to significant increases in α-carotene, β-carotene, and lycopene, which are important pigments that participate widely in various plant physiological processes and have strong antioxidant function, which is of great importance for maintaining cell homeostasis in adverse environments. Thus, metabolic manipulation of HMGR may constitute an alternative strategy for poplar breeding.

A previous study reported that overexpression of *BjHMGS* and improvement of *SlGPS* and *SlGGPPS* resulted in significantly higher carotenoid and vitamin E content in tomato fruits, which is likely indicative of cross-talk between the MVA and MEP pathways (Liao et al., 2018). In the current study, we also investigated how *PtHMGR* overexpression increases terpenoid content. Transcription levels of MVA and MEP-related genes were measured. We found that the transcript levels of *DXS*, *DXR*, *HDS*, and *HDR* in the MEP pathway, and the expression levels of *IDI*, *GPS*, *GPPS*, and *GGPPS*, were significantly elevated when compared to transgenic and WT plants. Transcript levels of *MVK* and *MVD* in the MVA pathway also increased significantly in transgenic plants. Together, these results suggest that overexpression of *HMGR* in the cytosol affects not only the expression of MVA-related genes, but also the transcript levels of MEP-related genes. *PtHMGR*-overexpressing poplars exhibited significantly higher α-carotene, β-carotene, and lycopene content, perhaps due to cross-talk between the MVA and MEP pathways. When *DXS* is over- or underexpressed in *Arabidopsis*, its transcript levels change according to endogenous isoprenoid content, suggesting a possible function for DXS in the regulation of isoprenoids biosynthesis. Surprisingly, *DXS* expression influenced endogenous ABA levels, suggesting that the early stages of ABA synthesis may partly contribute to ABA regulation (Estévez et al., 2001). In this study, *PtHMGR*-overexpressing poplars exhibited significantly higher transcript levels of *NCED* and *ZEP*, as well as improved endogenous ABA levels. Treatment of poplar with exogenous ABA resulted in increased *PtHMGR* expression. Therefore, HMGR not only regulates ABA biosynthesis, but is also regulated by ABA feedback. ABA is also an endogenous messenger that responds to a wide range of biotic and abiotic stresses in plants (Ton et al., 2009). ABA content is associated with many physiological processes, and the regulation of its biosynthesis is a key factor therein. We also observed an interesting phenomenon regarding the transcription levels of IAA-related genes. Although IAA is not a terpenoid, significant changes in the *IAA1*, *IAA18*, and *IAA19* transcript levels occurred, as did slight changes in the transcript levels of *IAA2* and *IAA6*.

In conclusion, we cloned a full-length gene encoding *PtHMGR*, which is involved in the biosynthesis of terpenoids. PtHMGR was functionally characterized and demonstrated to be the key entry point enzyme. Moreover, overexpression of *PtHMGR* changed the transcript levels of MVA-, MEP-, and downstream-related genes. Furthermore, significant positive correlations between *PtHMGR* expression and terpenoid content were revealed. These correlations further confirmed that PtHMGR could be one of the most important enzymes involved in the biosynthesis of terpenoids. Further studies to identify genes related to terpenoids would be useful not only for understanding terpenoid biosynthesis, but could also provide guidance for molecular breeding efforts.

## Data Availability Statement

All datasets for this study are included in the article/**Supplementary Material**.

## Author Contributions

HW carried out the experimental work and prepared the first draft of the manuscript and figures. CX, AM, and WS provided critical inputs for the study as well as during the preparation of the manuscript. QZ, CX, and DL designed the research and analyzed the results.

## Funding

This work was supported by the National Key Program on Transgenic Research (2018ZX08020002), the National Science Foundation of China (No. 31570650), and the Priority Academic Program Development of Jiangsu Higher Education Institutions.

## Conflict of Interest

The authors declare that the research was conducted in the absence of any commercial or financial relationships that could be construed as a potential conflict of interest.

## Supplementary Material

Figure S1 Nucleotide and deduced amino acid sequences of PtHMGR (XP_002300544.1). The complete deduced amino acid sequence is depicted in single-letter code beneath the corresponding nucleotide sequence. The initiation codon is boxed, and the termination codon is indicated with an asterisk.

Figure S2 Comparison of the deduced amino acid sequences of the conserved regions of PtHMGR and corresponding parts of other known HMGR proteins. The two conserved domains, the HMG-CoA binding domain and NADPH binding motif, are numbered and indicated with boxes.

Figure S3 Construction of the prokaryotic expression vectors for *PtHMGR* using *Sal*I and *Eag*I as restriction sites. **(A)** PET-28a as the prokaryotic expression vector for construction of PET-28a-*PtHMGR* using kanamycin as a screening marker. **(B)** pSUMO as the prokaryotic expression vector for construction of pSUMO-*PtHMGR* using kanamycin as a screening marker. **(C)** PET-32a as the prokaryotic expression vector for construction of PET-32a-*PtHMGR* using amphomycin as a screening marker. **(D)** PET-28a as the prokaryotic expression vector for construction of PET-28a-truncated *PtHMGR* using kanamycin as a screening marker.

Figure S4 Prokaryotic expression analysis of the truncated PtHMGR protein induced at 110 rpm at 10°C or 4°C. **(A)** Analysis of the truncated PtHMGR protein-induced at 110 rpm for 48 h at 10°C. Lane M: molecular mass marker; lane 1: negative control; lanes 2–4: colonies 1–3, respectively, induced with 1 mM IPTG. **(B)** Analysis of supernatant and precipitation induced at 110 rpm for 48 h at 10°C. Lane M: molecular mass marker; lane 1: negative control; lane 3: precipitation; lane 4: supernatant. **(C)** Analysis of the truncated PtHMGR protein-induced at 110 rpm for 72 h at 4°C. Lane M: molecular mass marker; lane 1: negative control; lanes 2–4: colonies 1–3, respectively, induced with 1 mM IPTG. **(D)** Analysis of the supernatant and precipitate induced at 110 rpm for 72 h at 4°C. Lane M: molecular mass marker; lane 1: negative control; lane 3: precipitate; lane 4: supernatant. **(E)** Purification of the truncated PtHMGR protein using the supernatant. Lane M: molecular weight marker; lane 1: supernatant of cell lysate; lane 2: flow-through; lanes 3–4: wash; lane 5: elution.

Figure S5 Detection of the truncated PtHMGR protein in the supernatant *in vitro* with 1 ml reaction mixture (2.5 mM K_2_HPO_4_, 5 mM KCl, 1 mM EDTA, 5 mM DTT, 1 mg/ml PtHMGR, 3 mM NADPH as a coenzyme, and 0.3 mM of HMG-CoA as a substrate, pH 7.2). **(A)** Total ion chromatogram of HPLC reaction products. The peak at retention time 2.2 was attributed to target production (MVA). **(B)** Extracted ion chromatography (XIC) analysis. **(C)** TOFMS analysis. The SCIEX TripleTOF 5600+ m/z value was 131.0710, consistent with MVA.

Figure S6 Analysis of the nucleotide sequences of PtHMGR-encoding genes in *P. trichocarpa*. Six *PtHMGR* genes were localized to chromosomes 1, 2, 4, 5, 9, and 11. The Potri. numbers of the *PtHMGR* genes obtained from the “Phytozome 12” data bank are as follows: (Potri.001G457000.1), (Potri.002G004000.1), (Potri.004G208500.1), (Potri.005G257000.1), (Potri.009G169900.1), and (Potri.011G145000.1).

Figure S7 The transformation process of Nanlin895 poplars. **(A)** Poplar leaf pieces and petioles infected by *Agrobacterium* EHA105 containing pGWB9-*PtHMGR* were cultured on regeneration medium supplemented with 200 mg/ml cefotaxime, 30 mg/ml kanamycin, 0.002 mg/L thidiazuron (TDZ), 0.5 mg/L N-6-benzyladenine (6-BA), 30 g/L sucrose, and 8 g/L agar at pH 5.8. **(B)** Putative shoots were cultured on bud elongation medium supplemented with 200 mg/ml cefotaxime, 20 mg/ml kanamycin, 0.001 mg/L TDZ, 0.2 mg/L 6-BA, 30 g/L sucrose, and 8 g/L agar, at pH 5.8. **(C)** Putative roots were cultured on root medium supplemented with 200 mg/ml cefotaxime, 10 mg/ml kanamycin, 30 g/L sucrose, and 8 g/L agar, at pH 5.8. **(D)** and **(E)** Transgenic lines were grown in soil. Bars represent lengths of (A and (B) 0.5 cm, (C) 1 cm, (D) 2 cm, and (E) 3 cm.

Figure S8 Molecular identification of poplar plants overexpressing *PtHMGR*. **(A)** Detection of the *PtHMGR* gene in transgenic lines and WT through PCR using the genome as a template, the primer of CaMV35S-F as the upstream primer, and the primer of ORF-*PtHMGR*-R as the downstream primer. Lane M: 2K molecular mass marker; lane 1: negative control; lanes 2–9: transgenic lines 1–8, respectively. **(B)** Detection of *PtHMGR* gene expression levels in transgenic lines and WT through qRT-PCR using cDNA as template. Three independent biological replicates were analyzed with three technical repeats. Vertical bars represent means ± SD (n = 3). *: significant difference at P < 0.05. **: significant difference at P < 0.01. ***: significant difference at P < 0.001.

Figure S9 The **(A)** ABA, **(B)** GA, and **(C)** IAA biosynthesis pathways in plants.

Figure S10 HPLC–MS/MS chromatogram of ABA standards and equations for ABA. HPLC–MS/MS chromatogram of standard ABA at **(A)** 0.1, **(B)** 0.2, **(C)** 0.5, **(D)** 2, **(E)** 5, **(F)** 20, (G) 50, and (H) 200 ng/mL concentrations, dissolved in methanol/0.1% formic acid. (I) Equations for the ABA standard curves.

Figure S11 HPLC-MS/MS chromatogram of GA3 standards and equations for GA3. HPLC-MS/MS chromatogram of standard GA3 at 0.1 **(A)**, 0.2 **(B)**, 0.5 **(C)**, 2 **(D)**, 5 **(E)**, 20 **(F)**, 50 **(G)**, and 200 **(H)** ng/ml concentrations, dissolved in methanol/0.1% formic acid. **(I)** Equations for the GA3 standard curves.

Figure S12 HPLC–MS/MS chromatogram of GA4 standards and equations for ABA. HPLC–MS/MS chromatogram of standard GA4 at **(A)** 0.5, **(B)** 2, **(C)** 5, **(D)** 20, **(E)** 50, and **(F)** 200 ng/ml concentrations, dissolved in methanol/0.1% formic acid. **(G)** Equations for the GA4 standard curves.

Figure S13 Plots of α-carotene, β-carotene, and lycopene standard curves based on values obtained from standards tested through HPLC. **(A)** α-carotene standard curve. **(B)** β-carotene standard curve. **(C)** Lycopene standard curve. **(D)** Equations of the α-carotene, β-carotene, and lycopene standard curves.

Table S1 Primer sequences used in this study.
