# Supplementary figures and images for: Characterization and Function of 3-Hydroxy-3-Methylglutaryl-CoA Reductase in Populus trichocarpa: Overexpression of PtHMGR Enhances Terpenoids in Transgenic Poplar
Source: Front Plant Sci. 2019 Nov 15;10:1476. doi: 10.3389/fpls.2019.01476 (PMC6872958; doi:10.3389/fpls.2019.01476)

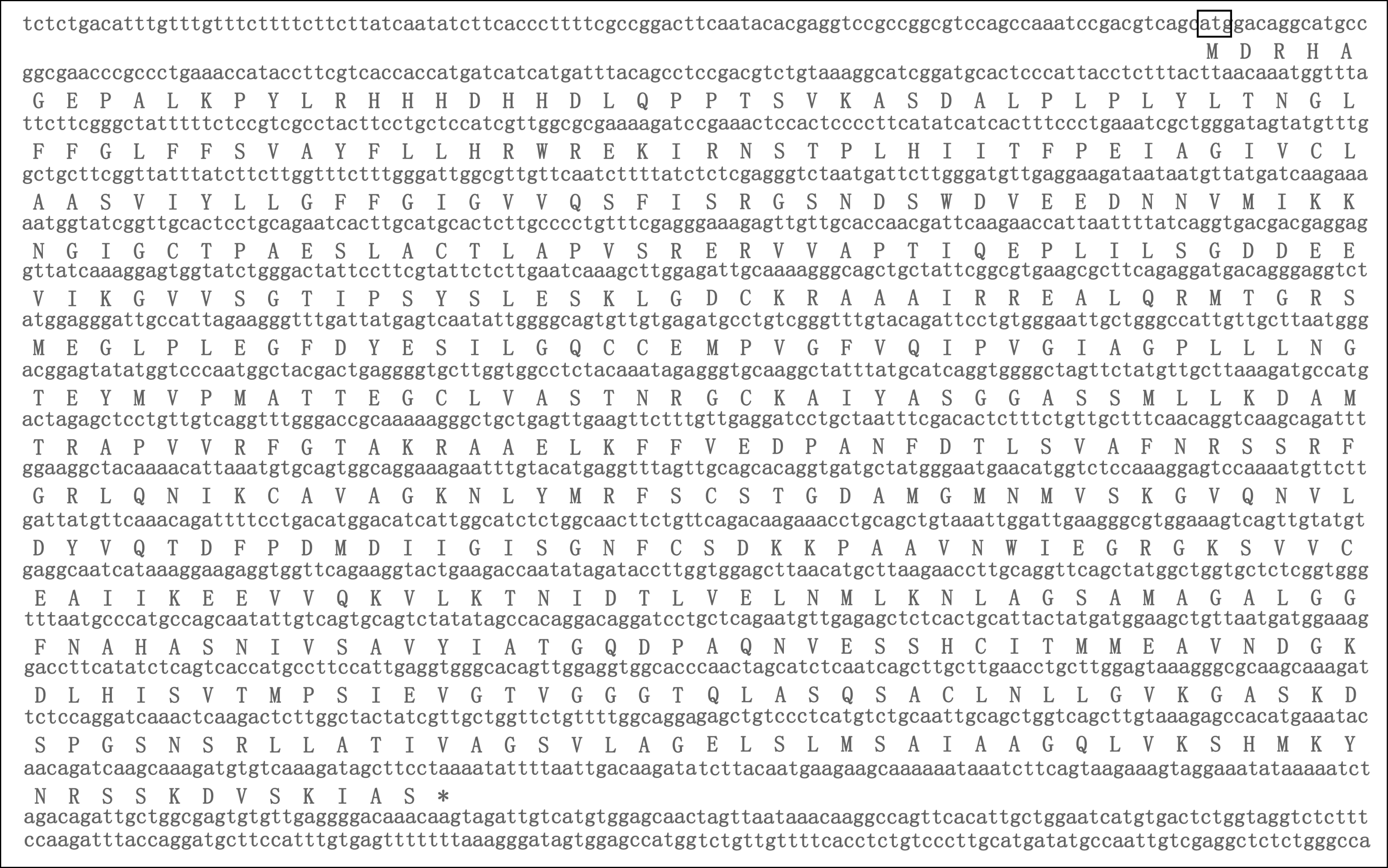

Supplement: Figure S1 — Nucleotide and deduced amino acid sequences of PtHMGR (XP_002300544.1). The complete deduced amino acid sequence is depicted in single-letter code beneath the corresponding nucleotide sequence. The initiation codon is boxed, and the termination codon is indicated with an asterisk. [file Image_1.jpeg]

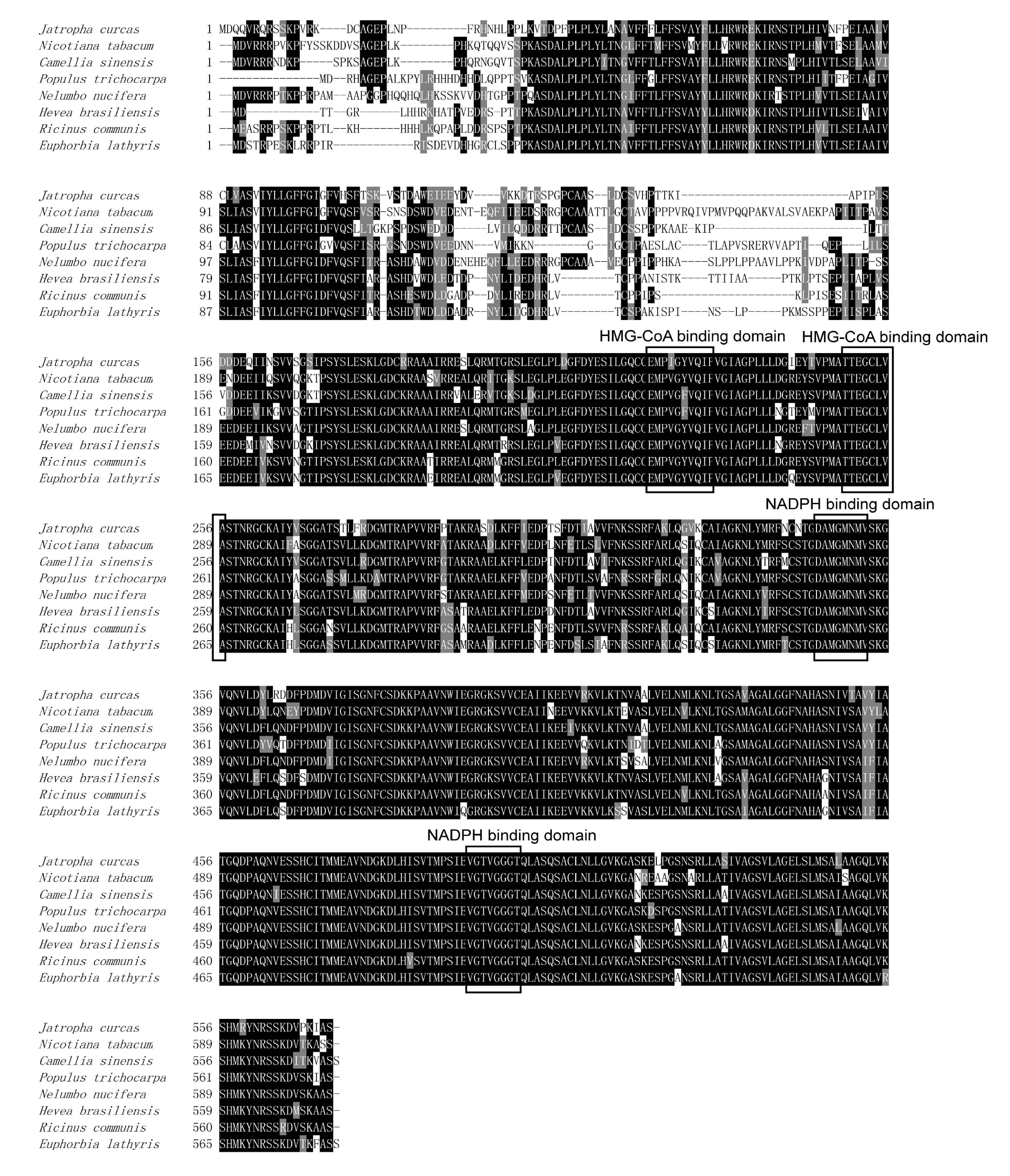

Supplement: Figure S2 — Comparison of the deduced amino acid sequences of the conserved regions of PtHMGR and corresponding parts of other known HMGR proteins. The two conserved domains, the HMG-CoA binding domain and NADPH binding motif, are numbered and indicated with boxes. [file Image_2.jpeg]

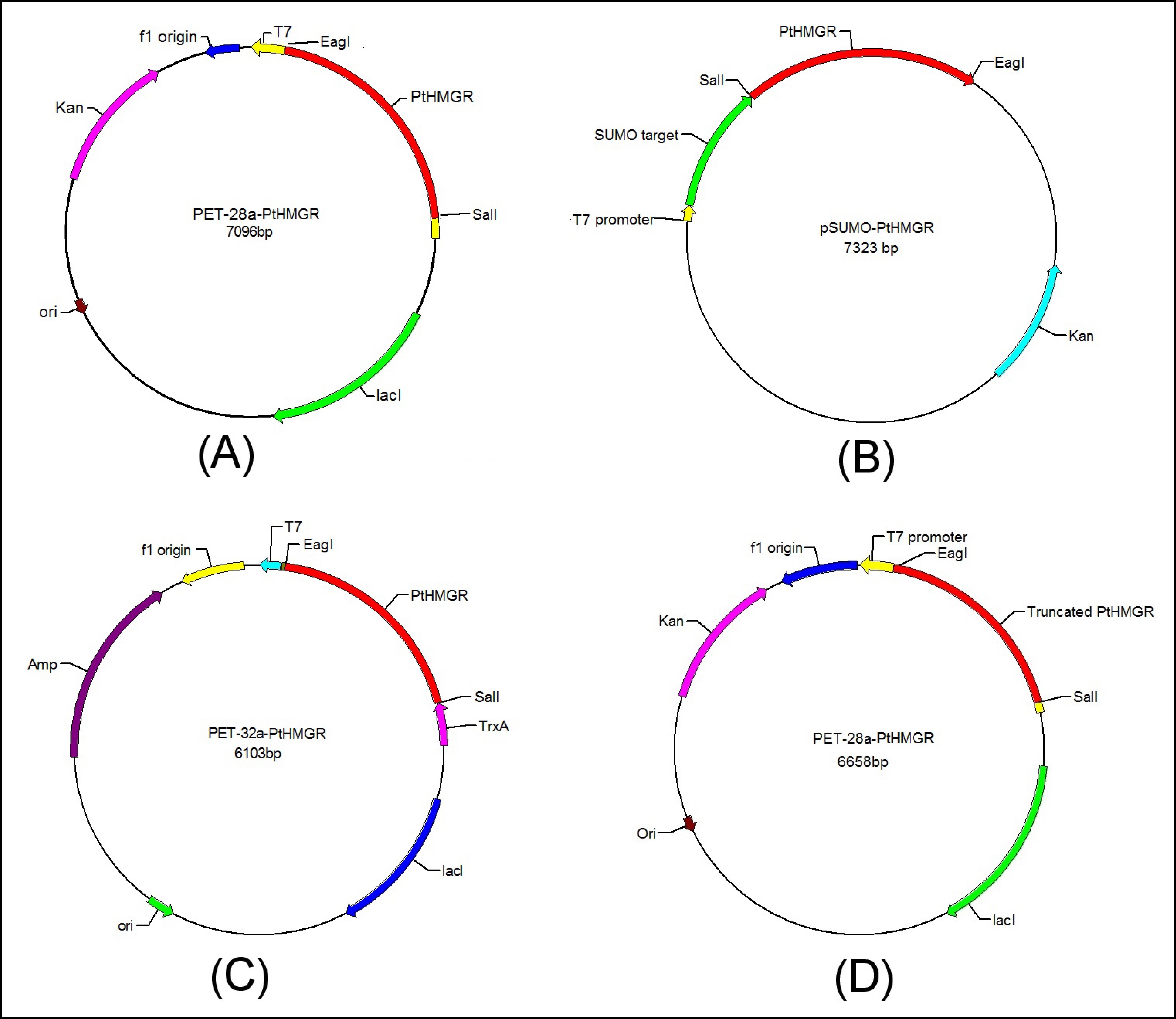

Supplement: Figure S3 — Construction of the prokaryotic expression vectors for PtHMGR using SalI and EagI as restriction sites. (A) PET-28a as the prokaryotic expression vector for construction of PET-28a-PtHMGR using kanamycin as a screening marker. (B) pSUMO as the prokaryotic expression vector for construction of pSUMO-PtHMGR using kanamycin as a screening marker. (C) PET-32a as the prokaryotic expression vector for construction of PET-32a-PtHMGR using amphomycin as a screening marker. (D) PET-28a as the prokaryotic expression vector for construction of PET-28a-truncated PtHMGR using kanamycin as a screening marker. [file Image_3.jpeg]

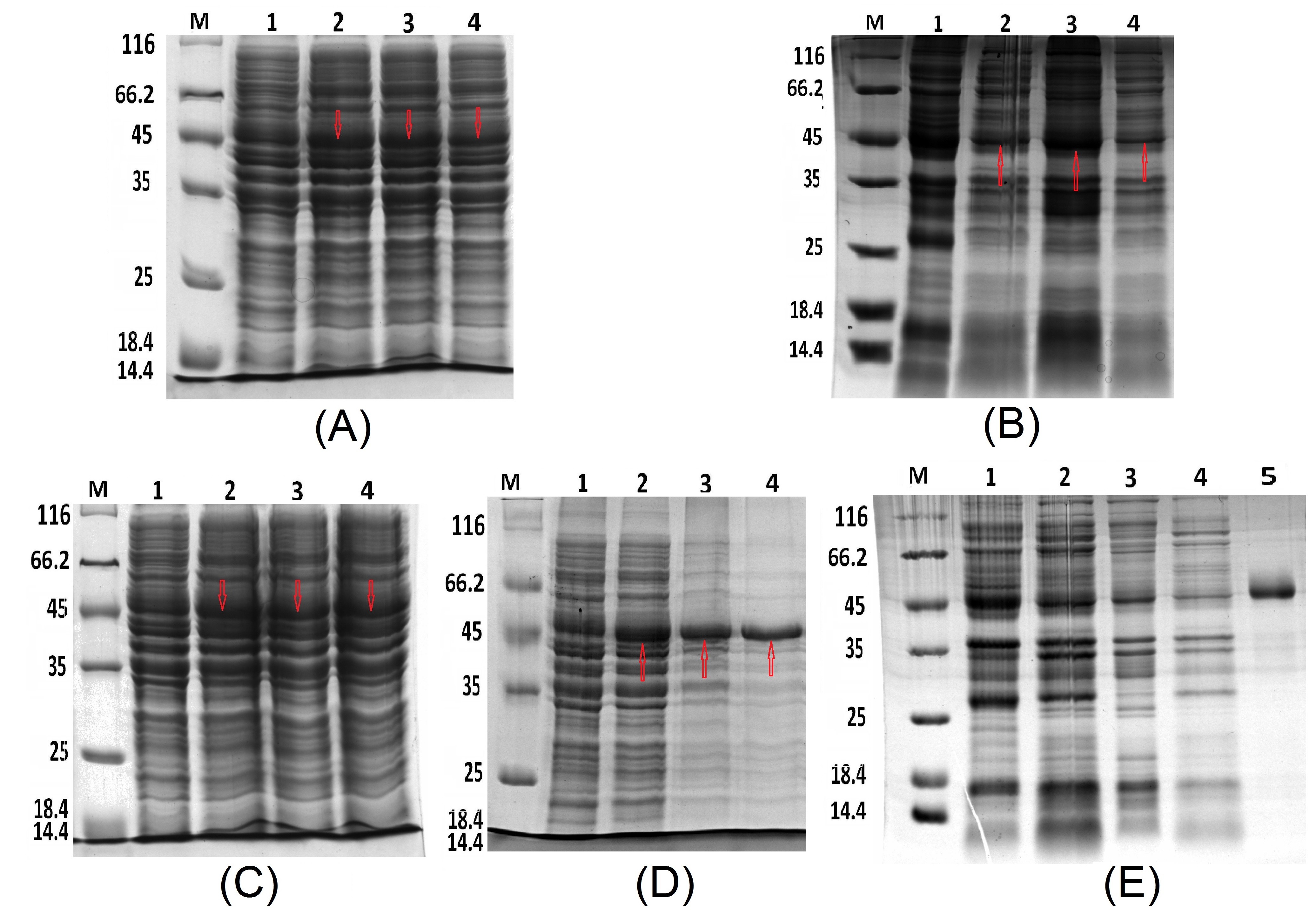

Supplement: Figure S4 — Prokaryotic expression analysis of the truncated PtHMGR protein induced at 110 rpm at 10°C or 4°C. (A) Analysis of the truncated PtHMGR protein-induced at 110 rpm for 48 h at 10°C. Lane M: molecular mass marker; lane 1: negative control; lanes 2–4: colonies 1–3, respectively, induced with 1 mM IPTG. (B) Analysis of supernatant and precipitation induced at 110 rpm for 48 h at 10°C. Lane M: molecular mass marker; lane 1: negative control; lane 3: precipitation; lane 4: supernatant. (C) Analysis of the truncated PtHMGR protein-induced at 110 rpm for 72 h at 4°C. Lane M: molecular mass marker; lane 1: negative control; lanes 2–4: colonies 1–3, respectively, induced with 1 mM IPTG. (D) Analysis of the supernatant and precipitate induced at 110 rpm for 72 h at 4°C. Lane M: molecular mass marker; lane 1: negative control; lane 3: precipitate; lane 4: supernatant. (E) Purification of the truncated PtHMGR protein using the supernatant. Lane M: molecular weight marker; lane 1: supernatant of cell lysate; lane 2: flow-through; lanes 3–4: wash; lane 5: elution. [file Image_4.jpeg]

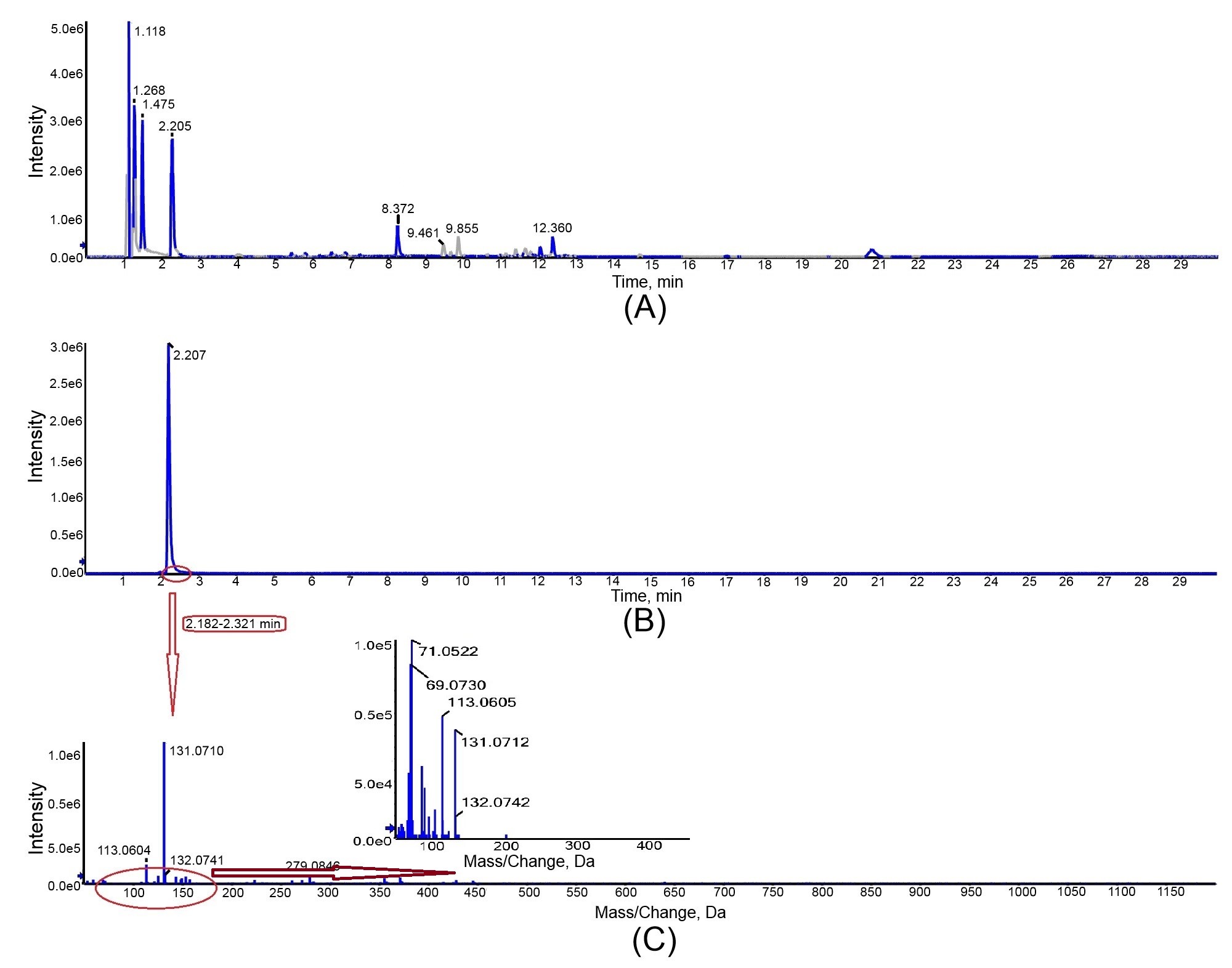

Supplement: Figure S5 — Detection of the truncated PtHMGR protein in the supernatant in vitro with 1 ml reaction mixture (2.5 mM K2HPO4, 5 mM KCl, 1 mM EDTA, 5 mM DTT, 1 mg/ml PtHMGR, 3 mM NADPH as a coenzyme, and 0.3 mM of HMG-CoA as a substrate, pH 7.2). (A) Total ion chromatogram of HPLC reaction products. The peak at retention time 2.2 was attributed to target production (MVA). (B) Extracted ion chromatography (XIC) analysis. (C) TOFMS analysis. The SCIEX TripleTOF 5600+ m/z value was 131.0710, consistent with MVA. [file Image_5.jpeg]

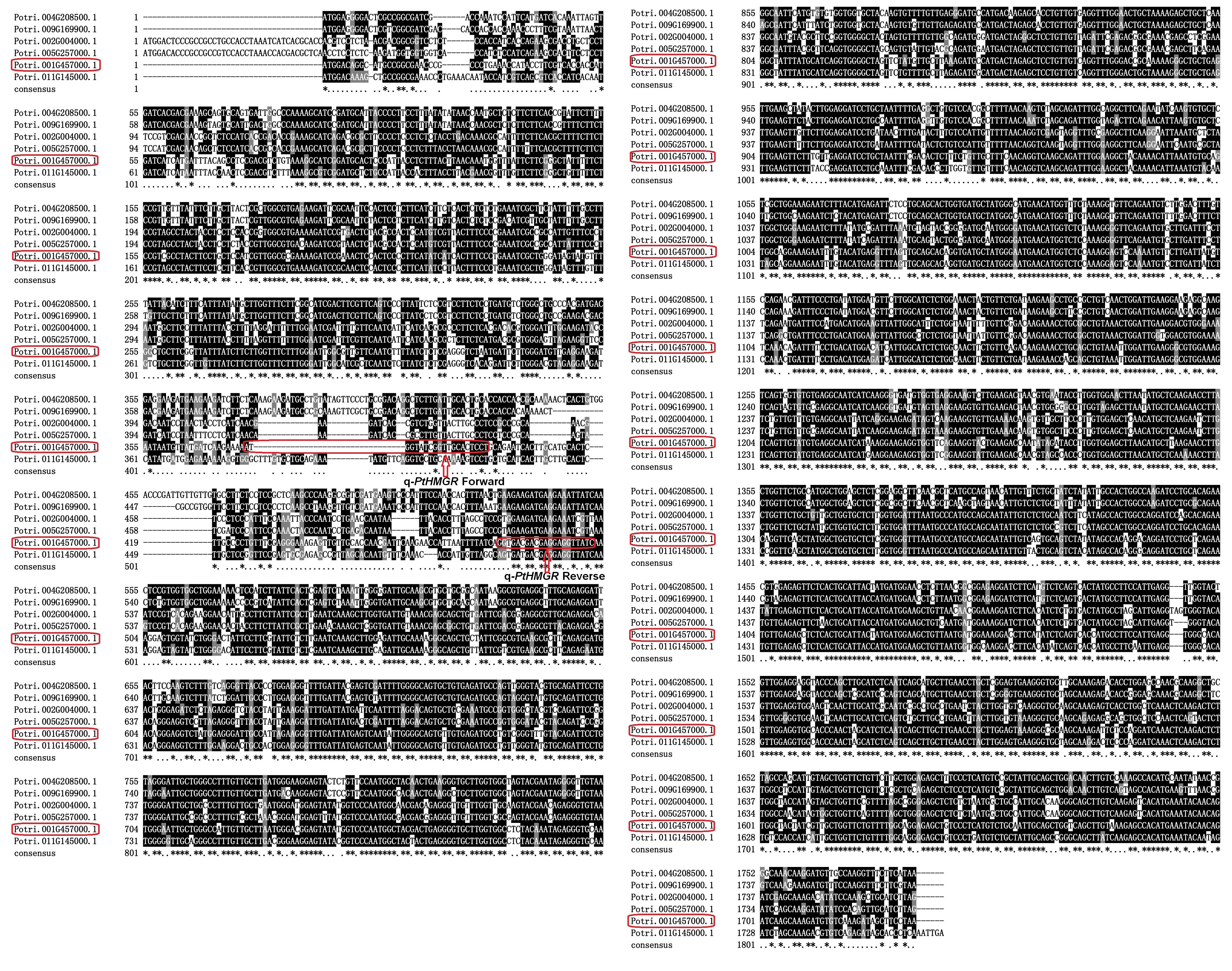

Supplement: Figure S6 — Analysis of the nucleotide sequences of PtHMGR-encoding genes in P. trichocarpa. Six PtHMGR genes were localized to chromosomes 1, 2, 4, 5, 9, and 11. The Potri. numbers of the PtHMGR genes obtained from the “Phytozome 12” data bank are as follows: (Potri.001G457000.1), (Potri.002G004000.1), (Potri.004G208500.1), (Potri.005G257000.1), (Potri.009G169900.1), and (Potri.011G145000.1). [file Image_6.jpeg]

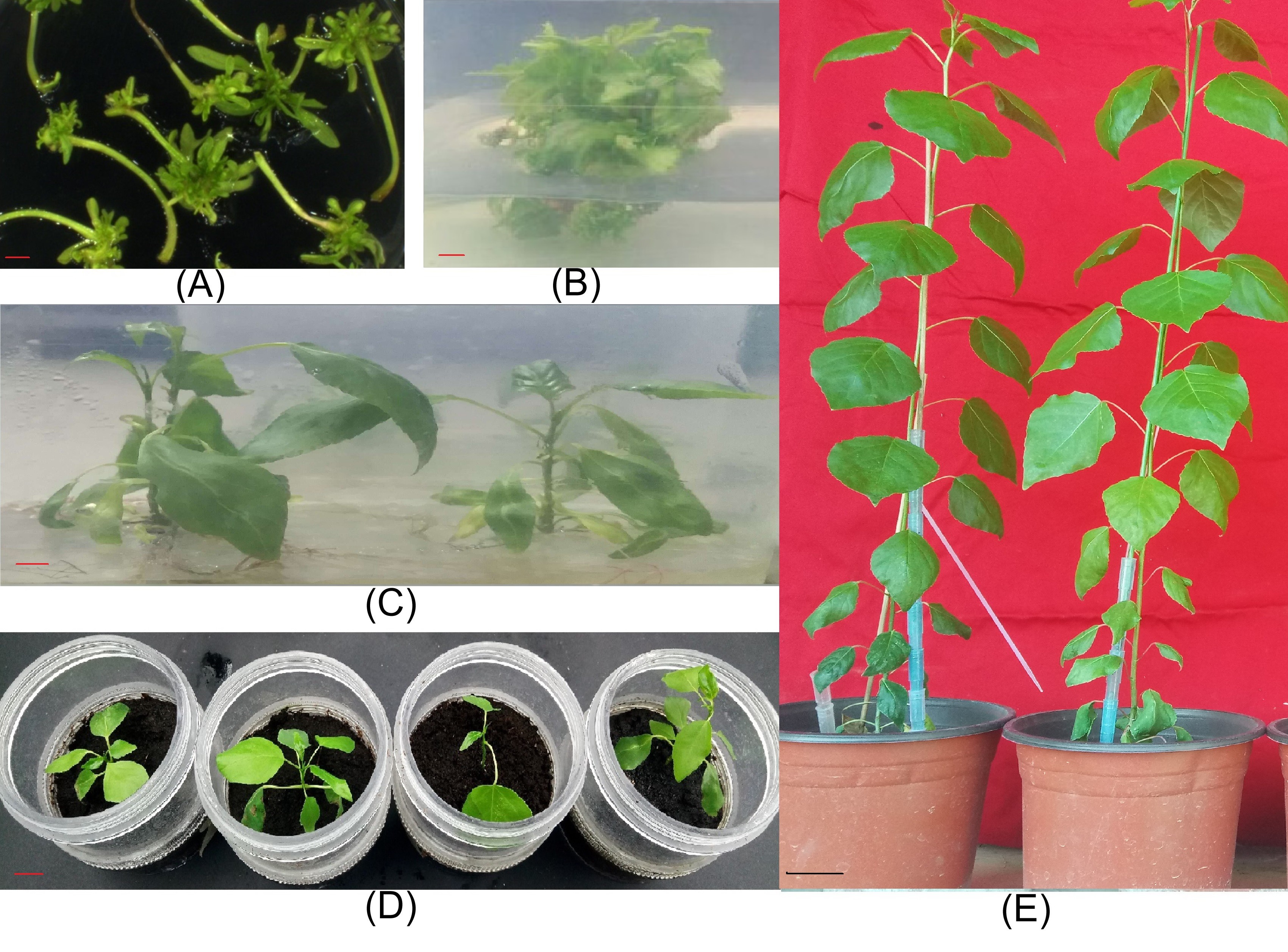

Supplement: Figure S7 — The transformation process of Nanlin895 poplars. (A) Poplar leaf pieces and petioles infected by Agrobacterium EHA105 containing pGWB9-PtHMGR were cultured on regeneration medium supplemented with 200 mg/ml cefotaxime, 30 mg/ml kanamycin, 0.002 mg/L thidiazuron (TDZ), 0.5 mg/L N-6-benzyladenine (6-BA), 30 g/L sucrose, and 8 g/L agar at pH 5.8. (B) Putative shoots were cultured on bud elongation medium supplemented with 200 mg/ml cefotaxime, 20 mg/ml kanamycin, 0.001 mg/L TDZ, 0.2 mg/L 6-BA, 30 g/L sucrose, and 8 g/L agar, at pH 5.8. (C) Putative roots were cultured on root medium supplemented with 200 mg/ml cefotaxime, 10 mg/ml kanamycin, 30 g/L sucrose, and 8 g/L agar, at pH 5.8. (D) and (E) Transgenic lines were grown in soil. Bars represent lengths of (A and (B) 0.5 cm, (C) 1 cm, (D) 2 cm, and (E) 3 cm. [file Image_7.jpeg]

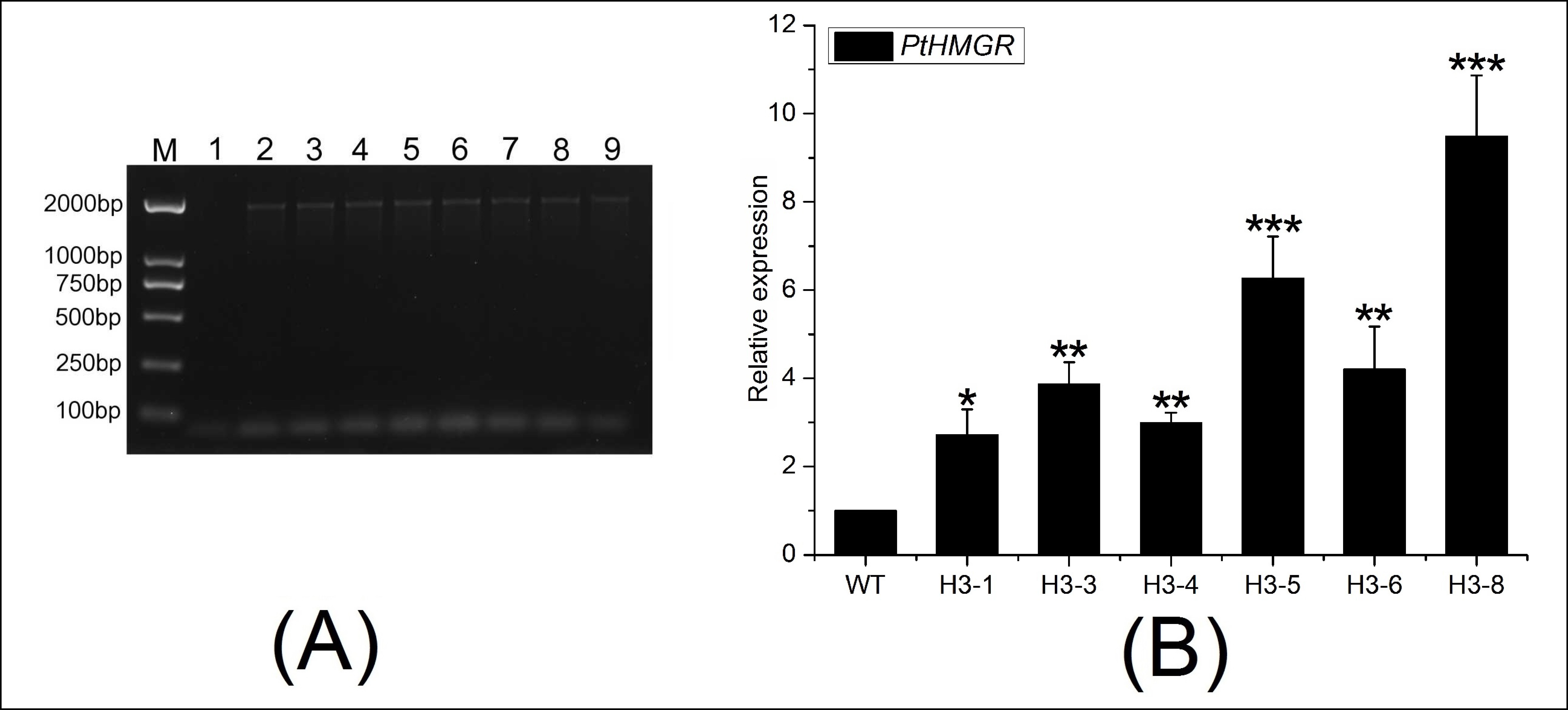

Supplement: Figure S8 — Molecular identification of poplar plants overexpressing PtHMGR. (A) Detection of the PtHMGR gene in transgenic lines and WT through PCR using the genome as a template, the primer of CaMV35S-F as the upstream primer, and the primer of ORF-PtHMGR-R as the downstream primer. Lane M: 2K molecular mass marker; lane 1: negative control; lanes 2–9: transgenic lines 1–8, respectively. (B) Detection of PtHMGR gene expression levels in transgenic lines and WT through qRT-PCR using cDNA as template. Three independent biological replicates were analyzed with three technical repeats. Vertical bars represent means ± SD (n = 3). *: significant difference at P < 0.05. **: significant difference at P < 0.01. ***: significant difference at P < 0.001. [file Image_8.jpeg]

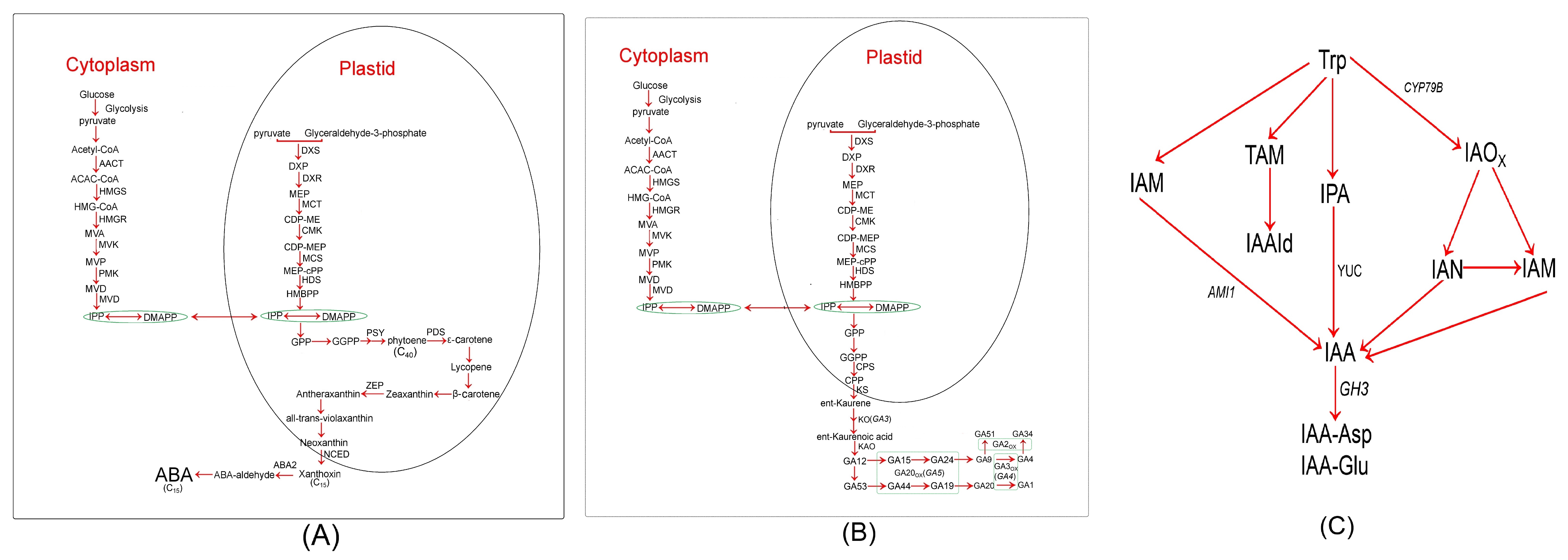

Supplement: Figure S9 — The (A) ABA, (B) GA, and (C) IAA biosynthesis pathways in plants. [file Image_9.jpeg]

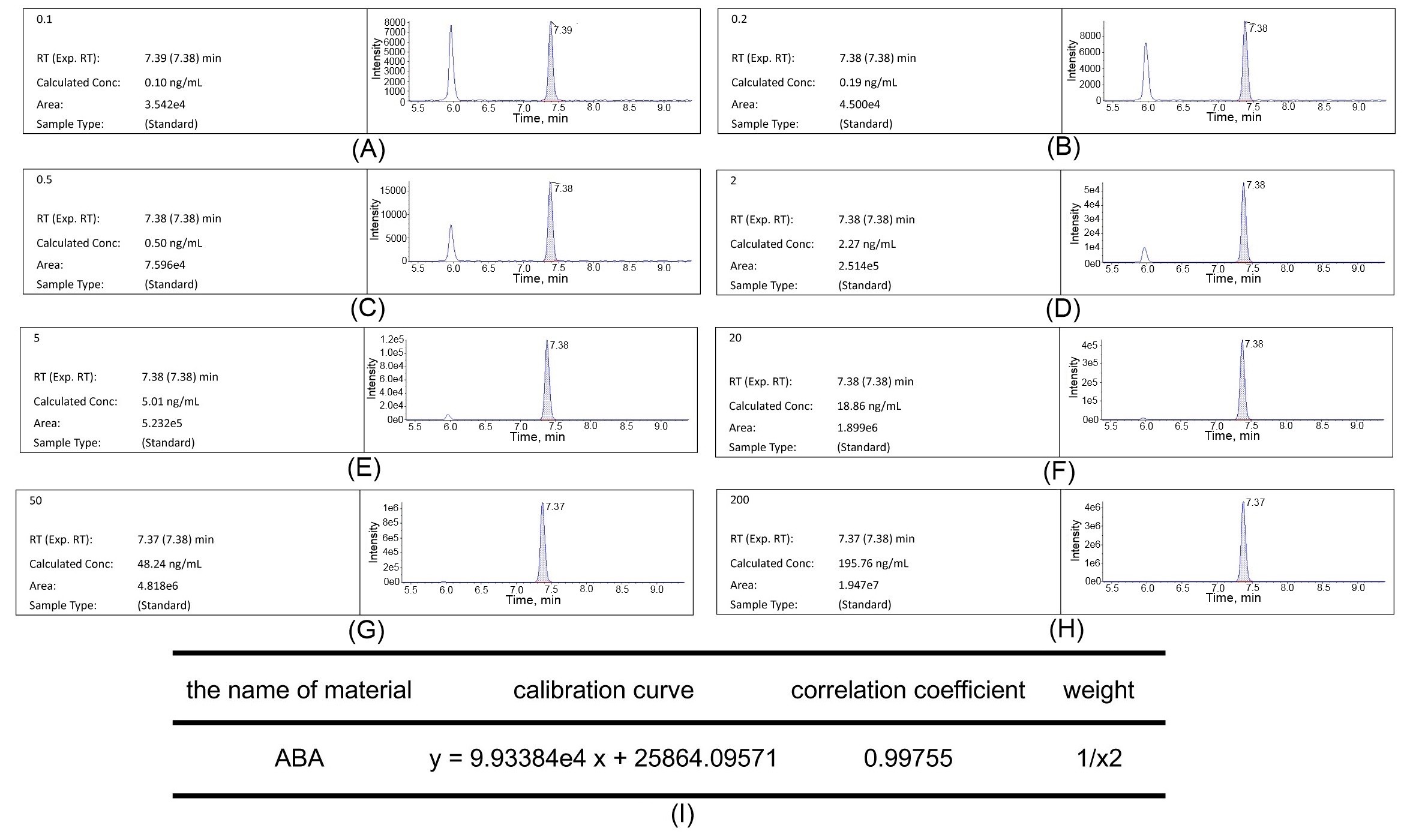

Supplement: Figure S10 — HPLC–MS/MS chromatogram of ABA standards and equations for ABA. HPLC–MS/MS chromatogram of standard ABA at (A) 0.1, (B) 0.2, (C) 0.5, (D) 2, (E) 5, (F) 20, (G) 50, and (H) 200 ng/mL concentrations, dissolved in methanol/0.1% formic acid. (I) Equations for the ABA standard curves. [file Image_10.jpeg]

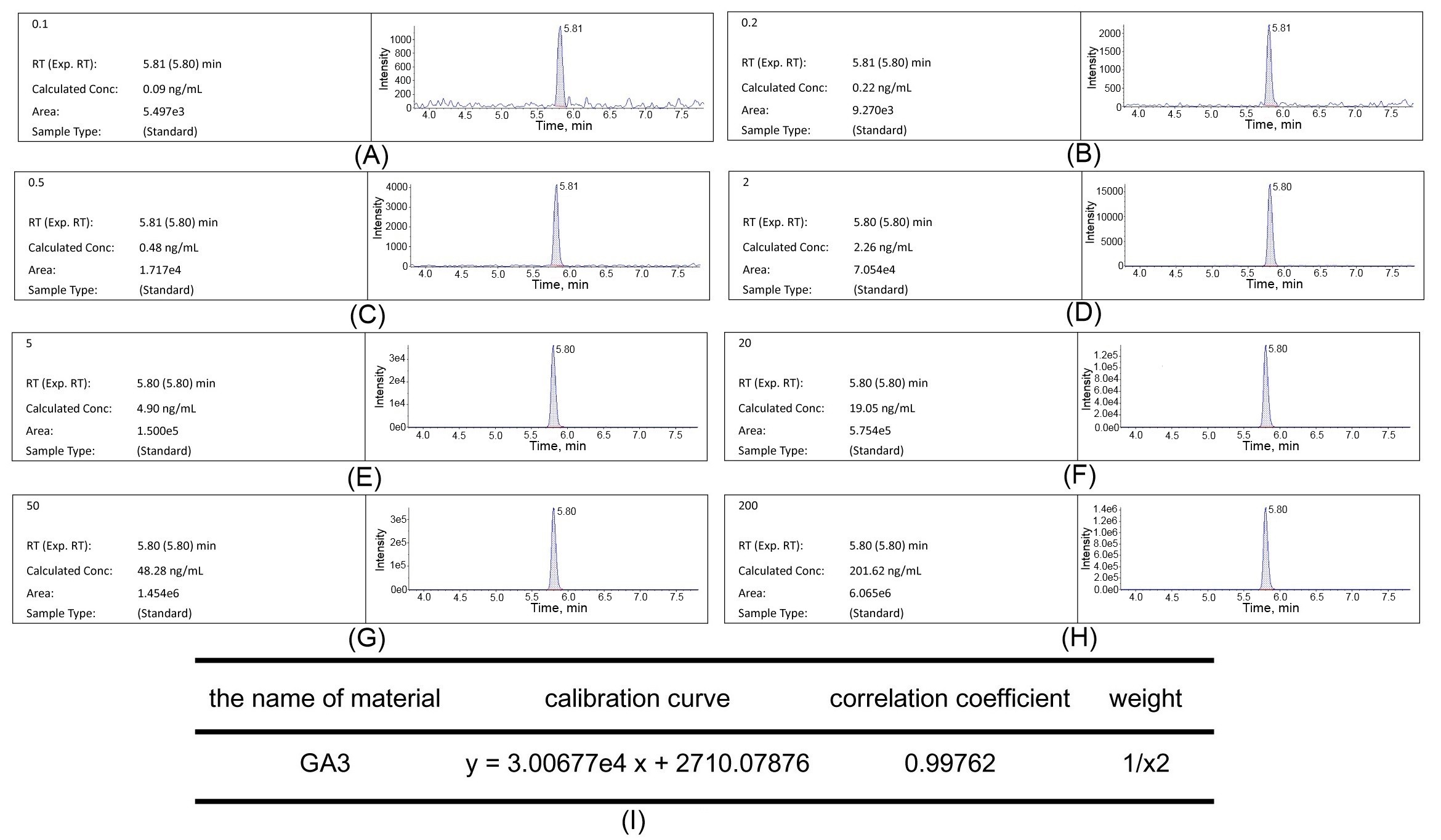

Supplement: Figure S11 — HPLC-MS/MS chromatogram of GA3 standards and equations for GA3. HPLC-MS/MS chromatogram of standard GA3 at 0.1 (A), 0.2 (B), 0.5 (C), 2 (D), 5 (E), 20 (F), 50 (G), and 200 (H) ng/ml concentrations, dissolved in methanol/0.1% formic acid. (I) Equations for the GA3 standard curves. [file Image_11.jpeg]

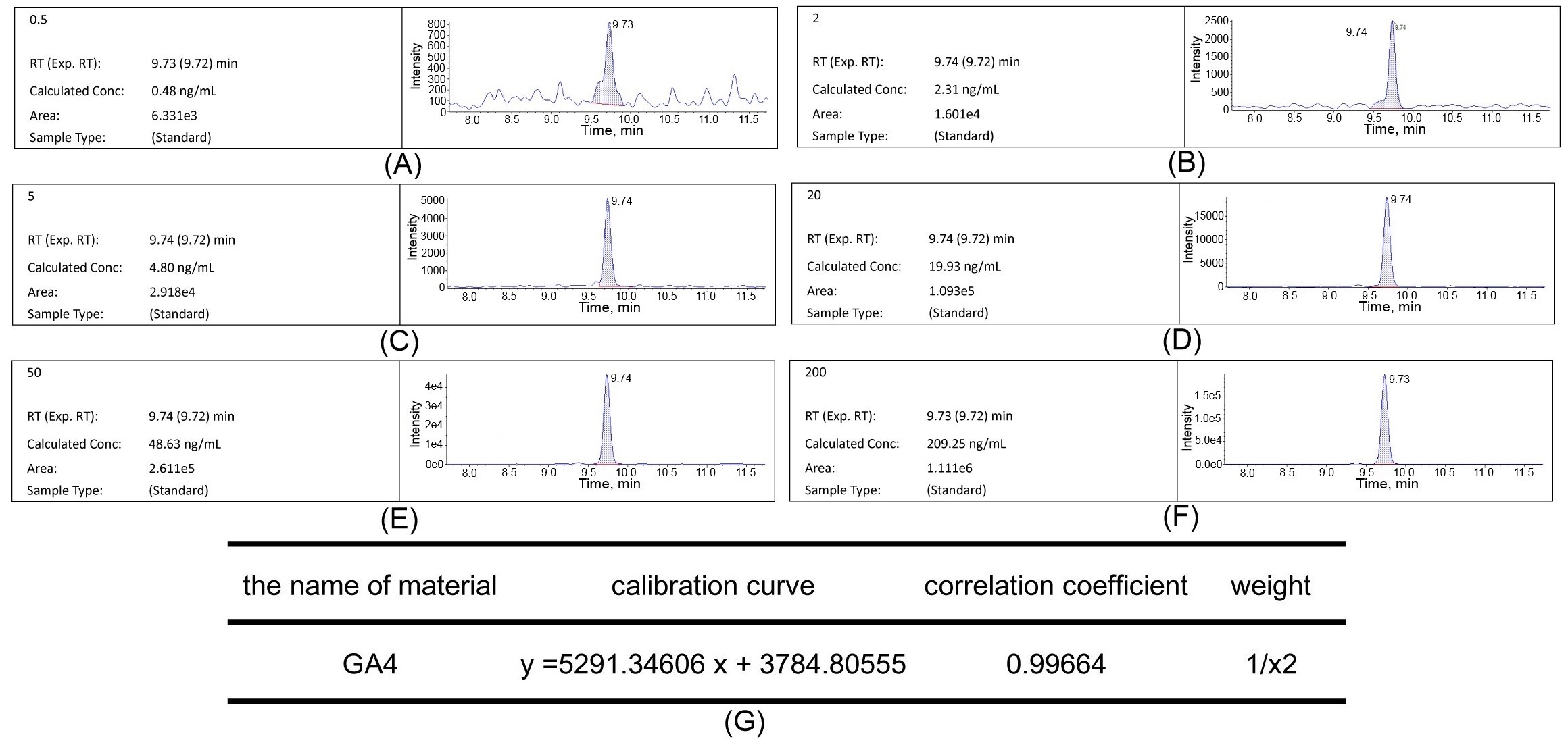

Supplement: Figure S12 — HPLC–MS/MS chromatogram of GA4 standards and equations for ABA. HPLC–MS/MS chromatogram of standard GA4 at (A) 0.5, (B) 2, (C) 5, (D) 20, (E) 50, and (F) 200 ng/ml concentrations, dissolved in methanol/0.1% formic acid. (G) Equations for the GA4 standard curves. [file Image_12.jpeg]

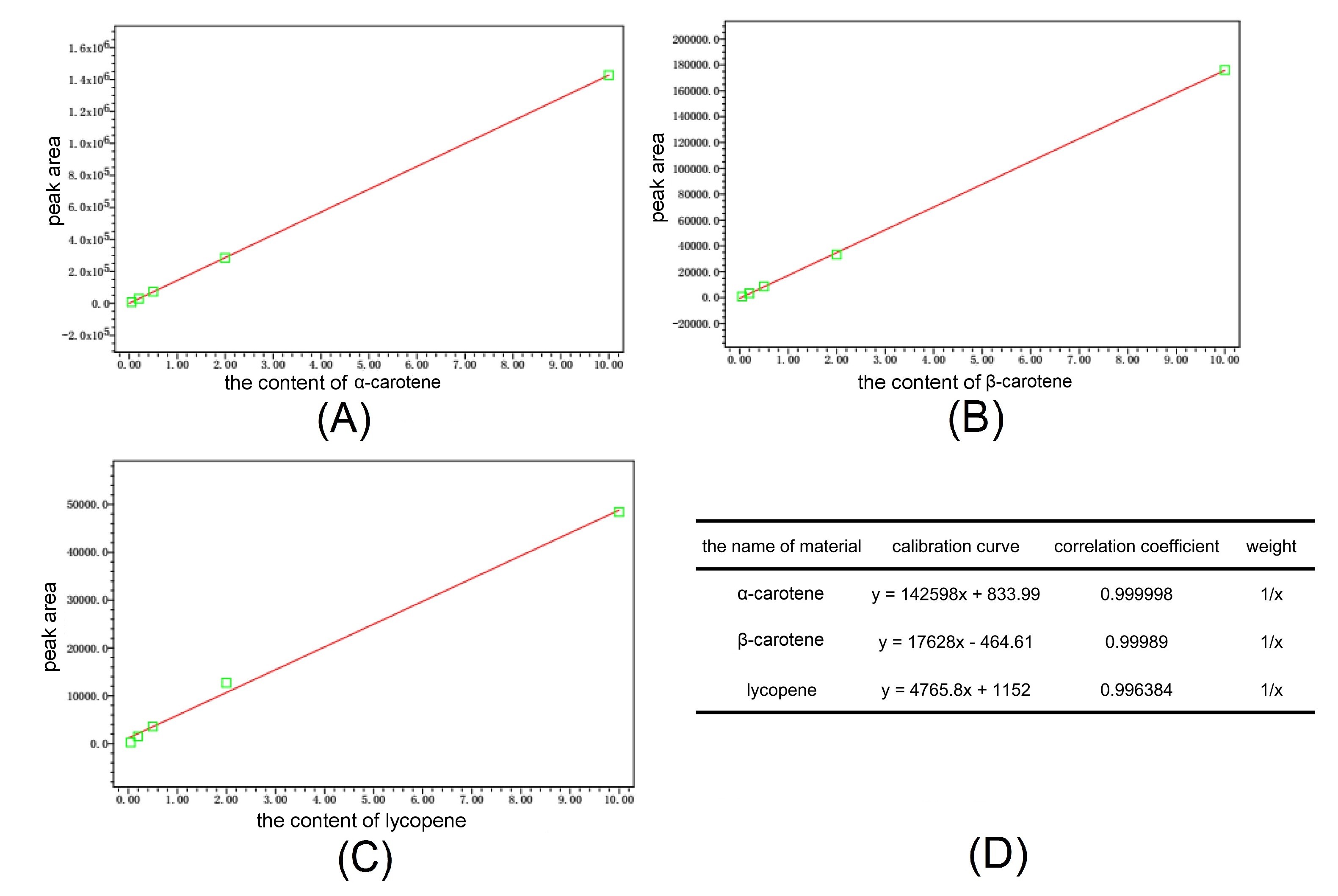

Supplement: Figure S13 — Plots of α-carotene, β-carotene, and lycopene standard curves based on values obtained from standards tested through HPLC. (A) α-carotene standard curve. (B) β-carotene standard curve. (C) Lycopene standard curve. (D) Equations of the α-carotene, β-carotene, and lycopene standard curves. [file Image_13.jpeg]
